# Structural variants are enriched in deleterious visible phenotypes in *Drosophila*

**DOI:** 10.1101/gr.281434.125

**Published:** 2026-07

**Authors:** Alejandra Samano, Matthew Musat, Mihir Junaghare, Asad Ahmad, Mehlum Ali, Sebastian Alves, Sreeram Pasupuleti, Jelisha Perera, Omar Saada, Brady Sabido, Trevor Smi1th, Sophie Walz, Mahul Chakraborty

**Affiliations:** Department of Biology, Texas A&M University, College Station, Texas 77843, USA

## Abstract

Genome structural variants (SVs) comprise a sizable portion of functionally important genetic variation, yet many evade discovery using short reads. Although long-read sequencing can reveal hidden SVs, their contribution to organismal trait variation remains unclear. To address this gap, we investigate the molecular basis of 50 classical phenotypes in 11 *Drosophila melanogaster* strains using highly contiguous de novo genome assemblies generated with Oxford Nanopore Technologies long reads. These assemblies enable construction of a pangenome graph with nucleotide-resolution maps of SVs, including complex rearrangements such as the interchromosomal inverted duplication Dp(2;4)*ey*^*D*^ and large tandem duplications at the *Bar* locus. We uncover new candidate causal mutations for 15 phenotypes and new molecular alleles for two mutations comprising tandem duplications, transposable element (TE) insertions, and indels. For example, the wing-vein phenotype *plexus* (*px*^*1*^) links to a 1.5 kb partial tandem gene duplication, and the century-old *Curved* (*c*^*1*^) wing phenotype links to a 7.5 kb DM412 retrotransposon disrupting the coding sequence of the muscle protein gene *Strn-Mlck*. We also identify a candidate intergenic enhancer for *Ablp*^eyD^, supported by CRISPR-Cas9, and uncover eight SV alleles of previously identified causal genes, including uncharacterized SVs underlying the extensively studied white and yellow phenotypes. Overall, 67.4% of genes causing phenotypic changes harbor candidate SVs >100 bp, whereas only 28% are expected based on euchromatic SVs. Together, our results indicate that SVs are strongly enriched among this class of large-effect, deleterious visible phenotypes in *Drosophila*.

Understanding the mutational basis of phenotypic differences between individuals or species is a fundamental puzzle in biology. Mutations that cause large or perceptible changes in phenotypes play an important role in adaptive evolution, agriculture, and medical genetics (Dittmar et al. 2016; Marian 2020). Although genome-wide association and linkage mapping studies have identified thousands of candidate variants, they often focus on small mutations, such as single-nucleotide polymorphisms (SNPs) and short indels in nonrepetitive regions, which are readily detected with short-read sequencing. Genome structural variants (SVs)—including duplications, transpositions, deletions, insertions, and inversions—alter more nucleotides and often exert stronger functional effects yet remain systematically undercharacterized owing to longstanding technical barriers (Alkan et al. 2011).

Population genomics studies across diverse species consistently show that SVs segregate at lower frequencies than SNPs, a pattern interpreted as evidence that SVs are more often deleterious and subject to stronger purifying selection (Chakraborty et al. 2019; Abel et al. 2020; Collins et al. 2020; Hämälä et al. 2021; Collins and Talkowski 2025; Samano et al. 2025). This interpretation is supported by case studies in which SVs are directly implicated in disease and severe phenotypes (Talkowski et al. 2012; Merker et al. 2018; Collins and Talkowski 2025), as well as by evidence that transposable element (TE) insertions disrupt gene function, alter regulation, and reduce fitness (Sorek et al. 2002; Hollister and Gaut 2009; Lee and Karpen 2017). However, the inference that SVs are generally deleterious largely derives from allele-frequency patterns rather than from direct analyses of phenotypic effects. As a result, despite extensive population genetic evidence suggesting that SVs disproportionately underlie harmful phenotypic changes, this hypothesis has rarely been tested using a defined, phenotypically diverse set of traits.

Visible phenotypic changes provide a powerful model for uncovering genotype–phenotype relationships (Sax 1923; Koornneef et al. 1983; Long et al. 1995; Doebley 2004). Historically, visible phenotypes formed the foundation of classical genetics: Early geneticists used them to construct genetic maps and uncover fundamental principles of inheritance (Bateson et al. 1905; Sturtevant 1913), including landmark discoveries from Mendel's pea experiments (Mendel 1996), Morgan's *Drosophila* work (Morgan 1910; Bridges 1922; Muller 1927), and McClintock's studies in maize (McClintock 1950). In particular, delineating mutations underlying morphological variation has helped illuminate evolution within and between species as well as elucidate genetic mechanisms of pathological conditions (Wild et al. 1997; Jeong et al. 2008; Chan et al. 2010; Imsland et al. 2012; Ghodsinejad Kalahroudi et al. 2014; Van't Hof et al. 2016). A systematic inquiry into the roles of SVs and SNPs in the molecular basis of morphological changes within a species can help elucidate the molecular properties of mutations underlying these phenotypic changes.

The model organism *Drosophila melanogaster* has an extensive collection of phenotype markers, many of which are deleterious to the organism. These mutations were collected based on their distinctive, easily scorable phenotypes rather than on prior knowledge of the underlying molecular basis, making them an unbiased set of large-effect variants whose causal mutations remain incompletely resolved. Although TEs are known contributors to several visible phenotypes (Green 1988; Sankaranarayanan 1988), no comprehensive investigation has quantified the contribution of SVs to this foundational genetic resource. Here, we investigate the molecular basis of 50 classical visible phenotypes by generating highly contiguous de novo genome assemblies for 11 *D. melanogaster* strains using Oxford Nanopore Technologies (ONT) long-read sequencing. We employed a pangenomic approach, constructing a graph-based representation of variation across the 11 assemblies relative to the ISO-1 reference. Using this comprehensive map of variation, we identify both known and previously uncharacterized candidate causal alleles, including duplications, TE insertions, complex rearrangements, and regulatory mutations.

## Results

### De novo genome assembly

**Figure 1.**
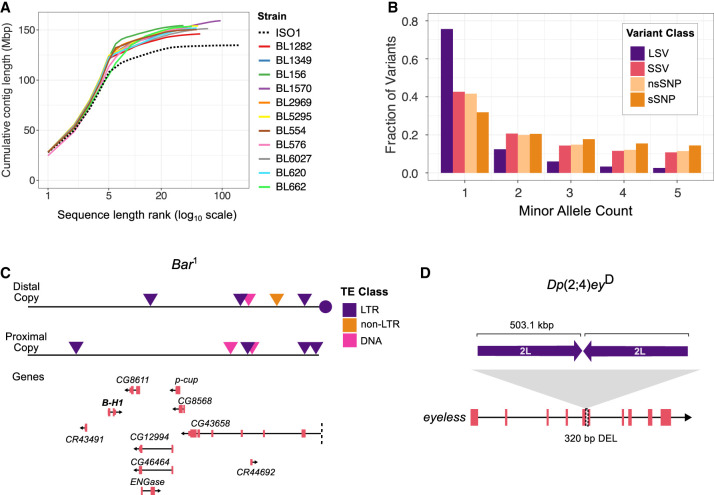
Genome assemblies reveal the structure and frequency of variants underlying visible phenotypes. (*A*) Contiguity plot comparing the ISO-1 release 6 contig-level reference assembly with the 11 de novo genome assemblies generated in this study. (*B*) Minor allele counts for large SVs (LSVs; >100 bp), small SVs (SSVs; 11–100 bp), nonsynonymous SNPs (nsSNPs), and synonymous SNPs (sSNPs). (*C*) Gene and TE content of the duplicated sequences at the *Bar* locus (ISO-1 coordinates X: 17,334,493–17,537,993), which contains nine complete genes and one truncated gene (*CG4368*). A *roo* element (circle) separates the two copies, consistent with the hypothesized mechanism of TE-mediated duplication. (*D*) The Dp(2;4)*ey*^*D*^ mutation is a translocation–duplication of a large segment of Chromosome 2L inserted into an exon of the *eyeless* gene on Chromosome 4, replacing 320 bp of coding sequence.

We collected deep ONT long-read coverage (average 90×, genome size G = 140 Mb) for 11 genomes carrying 50 visible mutations: 45 are spontaneous, four are caused by X-ray irradiation, and one is caused by a chemical mutagen (Supplemental Table S1). Although ONT long reads have an average accuracy of 85%–95%, the high coverage used here (55–141×) yields high consensus accuracy across genomic positions (Supplemental Table S2; Sereika et al. 2022; Kolmogorov et al. 2023). Consistent with this, base-level quality-value (QV) estimates derived from ONT-only error profiling (see Methods) range from 44.6 to 52.3 across assemblies, indicating high consensus accuracy (Table 1). Uniform long-read coverage across assemblies suggests an absence of large-scale assembly errors (Supplemental Fig. S1). All chromosome arms in our assemblies are represented by highly contiguous sequences (median assembly contig N50 = 23.1 Mbp), which include all of the euchromatin and a portion of pericentromeric heterochromatin in single contigs (Fig. 1A; Supplemental Fig. S2). However, balancer chromosomes in two strains (1570 and 6027) are fragmented owing to challenges in assembling heterozygous sequences with long, error-prone reads (Li and Durbin 2024). The number of dipteran Benchmarking Universal Single Copy Orthologs (BUSCOs) in these genomes (complete BUSCO scores 98.6–99.5) and contiguity are comparable to the reference genome ISO-1 (complete BUSCO score 98.8), further underscoring the high quality of the assemblies (Table 1; Supplemental Table S3).

**Table 1.** Assembly statistics

Strain name	Contigs			Scaffolds		Diptera complete BUSCOs	QV
	Length (Mb)	No. of contigs	N50 (Mb)	No. of scaffolds	N50 (Mb)		
1282	146.0	56	24.1	36	26.6	99.4	47.7
1349	151.2	49	24.2	28	29.4	99.4	46.5
156	154.3	36	23.8	18	29.3	99.4	49.0
1570	159.2	96	21.8	68	28.8	99.4	47.9
2969	150.1	34	23.8	17	29.7	99.4	44.6
5295	154.1	53	24.3	32	29.9	99.4	49.2
554	150.4	51	24.1	28	28.8	99.4	48.1
576	151.8	44	21.7	23	29.1	99.5	50.8
6027	151.4	69	23.9	49	27.9	98.6	48.6
620	151.9	51	24.3	29	27.6	99.4	52.3
662	153.5	45	18.5	21	29.0	99.4	48.2

### Landscape of genetic variation in 11 genomes

We identified mutations by comparing each genome assembly to the ISO-1 reference genome (Hoskins et al. 2015). To map genetic variation, we constructed a pangenome graph that captures all mutation classes (Hickey et al. 2023). Unlike traditional approaches that compare genomes to a single linear reference, a pangenome graph represents sequences as nodes in a network, enabling identification of both shared and unique variants, including SNPs, small indels, and SVs across all genomes (Eizenga et al. 2020; Sirén et al. 2021). Although we also performed pairwise genome alignments and read mapping to validate genotypes at candidate loci, the pangenome graph provides a comprehensive and accurate view of genetic variation. Focusing on euchromatic regions of the five major chromosome arms (2L, 2R, 3L, 3R, and X), we identified 53,337 small structural variants (SSVs; 11–100 bp) and 11,587 large structural variants (LSVs; >100 bp) across the 11 genomes. Of the LSVs, 7156 were associated with TEs. In total, 1.64 million SNPs were detected, of which 5.2% are located in coding exons.

We examined the minor allele-frequency distribution of LSVs, SSVs, nonsynonymous SNPs (nsSNPs), and synonymous SNPs (sSNPs). LSVs are significantly skewed toward lower frequencies than nsSNPs, a pattern likely driven by TEs (*P*-value < 2.2 × 10^−16^, χ^2^ test between frequency distributions of LSVs and nsSNPs) (Fig. 1B). Many of the identified LSVs are TE insertions, and their low allele frequencies reflect both invasion dynamics and fitness consequences in *Drosophila*. Newly invading TE families produce large numbers of young insertions that initially segregate at low frequency (Kofler et al. 2015; Pianezza et al. 2025), and because most TE insertions are mildly to strongly deleterious, purifying selection further maintains these variants at low population frequencies (Charlesworth and Langley 1989; Petrov et al. 2011; Cridland et al. 2013). Although SSVs also showed a skew toward lower frequencies, their distribution is more similar to that of nsSNPs. These patterns align with previous population genomics studies, which suggest that SVs are subject to stronger purifying selection, likely owing to their more deleterious effects (Cridland et al. 2013; Chakraborty et al. 2019; Samano et al. 2025).

### Assembly of large, complex SVs

Large and repetitive SVs are often difficult to resolve at the molecular level (Treangen and Salzberg 2011). To assess the capacity of our assemblies to characterize such mutations, we analyzed two visible mutations known to be linked to large duplications. Strain 2969 carries the *Bar*^*1*^ allele, an X-linked mutation that causes a slit-eye phenotype in males and homozygous females (Tice 1914). Classical genetic analyses (Sturtevant 1925; Muller 1936), followed by molecular cloning and sequencing (Tsubota et al. 1989; Miller et al. 2016), demonstrated that the *Bar*^1^ allele arose by unequal crossing over between homologous *roo* elements at cytological positions 16A1 and 16A7 on different chromatids. This results in the tandem duplication of the 16A1-7 sequence with a single *roo* element retained between the duplicates, marking the site of recombination (Supplemental Fig. S3). Consistent with this model, our assembly of strain 2969 captures both copies of the 203.5 kb region between positions 16A1 and 16A7. The breakpoints match prior studies and include a complete *roo* element between the two copies, supporting the TE-induced duplication model (Supplemental Fig. S4). The duplicated segment contains seven complete protein-coding genes, including *BarH1*, which is associated with the *Bar*^1^ eye phenotype (Kojima et al. 1993), as well as two long noncoding RNAs (lncRNAs), and one truncated gene (Fig. 1C). Notably, the two copies show substantial sequence divergence, including TE insertions unique to one copy (Fig. 1C). We also analyzed strain 662, which carries the Dp(2;4)*ey*^*D*^ mutation, an X-ray-induced translocation–duplication resulting in reduced or absent eyes (Hochman et al. 1964). Consistent with earlier cloning experiments (Kronhamn et al. 2002), we identified a 503.1 kb sequence from Chromosome 2L that was duplicated, with one copy inverted and inserted into the *eyeless* gene on the fourth chromosome (Fig. 1D; Supplemental Fig. S5). This translocation disrupts a coding exon and removes 320 bp of coding sequence from *ey*. In contrast to the *Bar*^*1*^ duplicates, the duplicated sequences of the Dp(2;4)*ey*^*D*^ mutation show fewer sequence differences.

### Discovery of previously uncharacterized mutations

Strains carrying the *Bar*^*1*^ and Dp(2;4)*ey*^*D*^ mutations were included in this study owing to their known association with large genomic rearrangements. In contrast, the other strains were selected without prior consideration of the molecular basis of their visible phenotypes. We first examined the molecular nature of mutations in genes previously linked to phenotypes, such as the *white* eye color mutation, as hidden SVs can mislead inferences about the causal mutations underlying phenotypic changes (Chakraborty et al. 2019; Ebert et al. 2021; Fadaie et al. 2021). For each phenotype, we identified candidate mutations as variants predicted to disrupt coding regions or located in putative regulatory elements (e.g., promoters, enhancers, UTRs) that could affect gene expression and were unique to strains with the phenotype. If the previously documented mutation was absent, we checked for another disruptive mutation in the same gene. Among the 50 phenotypes examined, we confirmed the previously characterized causal mutation for 33. We identified 18 uncharacterized mutations in total, including two that co-occurred with the previously reported causal mutation and one that contradicted the documented allele for that genotype reported on FlyBase (Öztürk-Çolak et al. 2024). For 15 phenotypes with no prior molecular characterization, we used our pangenome graph to identify candidate mutations.

**Figure 2.**
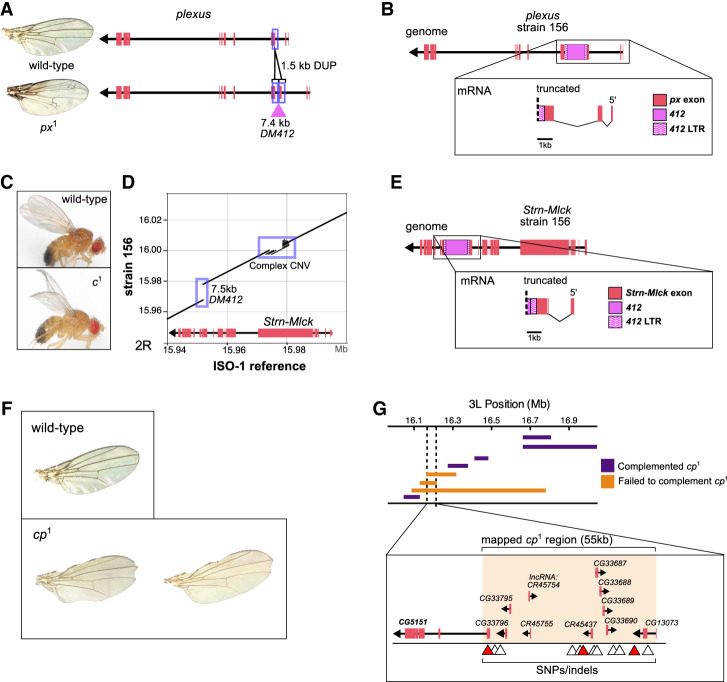
Structural variants underlying the *plexus* and *curved* visible phenotypes and their transcriptional consequences. (*A*) Wing phenotypes and genomic organization of the *plexus* (*px*¹) locus. Compared to the wild-type, *px*¹ flies show excess wing-vein patterning. The *px*¹ allele is associated with a 1.5 kb tandem duplication of a partial exon and a 7.4 kb insertion of the LTR retrotransposon, *DM412*. (*B*) Genomic structure and transcript model of *plexus* in strain 156, supported by ONT direct mRNA sequencing. The *DM412* insertion disrupts the gene, producing a truncated, chimeric transcript that includes transposon-derived sequence and lacks downstream exons. (*C*) Wing phenotypes of wild-type and *c*^1^ flies. (*D*) Genome alignment at the *Strn-Mlck* locus underlying the *curved* (*c¹*) mutation. Relative to the ISO-1 reference, strain 156 carries a 7.5 kb *DM412* insertion and a complex copy-number variant in *Strn-Mlck*. (*E*) Genomic and transcript models of *Strn-Mlck* in strain 156, supported by direct mRNA sequencing. The DM412 insertion truncates the transcript, resulting in loss of distal coding exons and incorporation of transposon sequence. (*F*) Wing phenotypes in wild-type and *cp*¹ flies. *cp*¹ mutants show variable wing notching patterns. (*G*) Complementation mapping with overlapping deletions narrowed the *cp*¹ candidate region to a 55 kb interval on Chromosome 3L (3L: 16,162,336–16,217,328; ISO-1 r6) containing *CG5151*, a gene implicated in wing development. Within this region, 15 SNPs and small indels unique to *cp*¹ strains were identified (triangles), with three candidates highlighted (red) based on their location within *CG5151* or overlap with wing disc ATAC-seq peaks.

The mutation *px*^1^ is associated with increased wing veins at the wing margins and tips. However, the mutation responsible for this gene is yet to be characterized. We found a 1.5 kb tandem duplication that copies the 5′ region of the first coding exon of *plexus* (*px*), with the duplicated segments separated by a 7.4 kb *DM412* retrotransposon (Fig. 2A). Long-read direct RNA-seq data reveal that this insertion produces a truncated, chimeric *px* transcript that includes 462 bp of transposon-derived sequence and terminates prematurely (Fig. 2B; Supplemental Fig. S6). This truncation removes the cysteine-rich and Q/QREKE motifs thought to be essential for Px function (Matakatsu et al. 1999). Loss of functional *plexus* is therefore likely to impair repression of wing-vein development, providing a mechanistic explanation for the ectopic wing-vein phenotype observed in *px*¹ mutant wings.

For two of the 15 uncharacterized phenotypes, the underlying genes had not yet been mapped in the *D. melanogaster* genome, so we combined our comprehensive variant map with additional genetic mapping experiments to identify candidate mutations for the phenotypes. The *curved* (*c*¹) mutation, which produces a characteristic curved wing phenotype (Fig. 2C), has not been mapped to the genome sequence (Supplemental Fig. S7). Previous studies based on deficiency maps narrowed the candidate region to 10 genes, with *Strn-Mlck* proposed as the most likely target because mutations in this gene produce wing phenotypes similar to those of *curved* (Rodriguez 2004; Kahsai and Cook 2018). In strain 156, we identified a 7.5 kb *DM412* LTR retrotransposon insertion within an exon of *Strn-Mlck*, together with nearby copy-number variation (CNV) in a region enriched for PEVK and SAIDE repeats in the protein (Fig. 2D). Long-read direct RNA-seq data demonstrate that the *DM412* insertion generates a truncated, chimeric *Strn-Mlck* transcript containing 673 bp of transposon-derived sequence before premature termination (Fig. 2E; Supplemental Fig. S8). Notably, this *DM412* insertion is absent from strains lacking the *c*¹ phenotype but is present in six additional *c*¹ strains (Supplemental Table S4), further supporting a causal link between the TE insertion and the curved wing phenotype. No other genes within the mapped interval harbor obvious disruptive mutations, implicating *Strn-Mlck* as a likely candidate underlying the *c*¹ phenotype. Definitive confirmation would require additional functional tests, such as targeted CRISPR-based disruption or allele replacement.

A clipped wing phenotype, *clipped* (*cp*^1^) (Fig. 2F), was mapped to a 3-Mbp region on Chromosome 3L. We further narrowed the region by crossing flies carrying the *cp*^1^ mutation with deficiency lines (see Methods), reducing it to a 55 kb window overlapping eight protein-coding genes and three lncRNAs (Fig. 2G; Supplemental Fig. S9; Supplemental Table S5). One of these genes, *CG5151*, has been shown to play an important role in wing development. Knockdown of the gene in the posterior imaginal wing disc results in wing notching similar to that observed in *cp*^1^ mutants (Bageritz et al. 2019). To identify candidate mutations underlying the *clipped* phenotype, we used our pangenome graph to detect variants shared among strain 620 and six additional *cp*^1^ mutant strains but absent from non-*clipped* strains. We identified 15 shared SNPs and small indels within the 55 kb interval harboring the *cp*^1^ locus (Fig. 2G; Supplemental Table S6). Notably, one 7 bp indel disrupts a predicted transcription factor binding site (TFBS) in CG5151, whereas an additional indel and one SNP fall within wing disc ATAC-seq peaks, indicating localization to regions of open chromatin. Although any of these variants could potentially contribute to the *clipped* phenotype, additional functional experiments will be required to determine causality. Importantly, we detected no shared SVs within the mapped interval across *cp*¹ strains. We therefore classify *clipped* as being associated with small variants rather than SVs. Thus, six phenotypes among the 15 without a candidate mutation were associated with SVs, and we infer the rest are caused by SNPs or indels involving 10 or fewer nucleotides.

In addition to the 50 mutations analyzed in this study, we investigated a candidate mutation underlying the unmapped *Abnormal leg pattern* (*Ablp*^eyD^) allele. This phenotype is associated with the X-ray-induced Dp(2;4)*ey*^*D*^ translocation duplication, which was specifically included to evaluate our ability to assemble and analyze large, repetitive mutations; therefore, it was not counted among the set of 50 phenotypes. *Ablp*^eyD^ was previously mapped to a 90 kb region on Chromosome 2L corresponding to the source of the sequence translocated onto Chromosome 4 in the Dp(2;4)*ey*^*D*^ mutation. Using the pangenome graph, we examined this 90 kb interval for candidate mutations shared between strain 662 and eight additional strains carrying the *Ablp*^eyD^ allele (see Methods). However, no SVs, SNPs, or indels within this region were uniquely shared across the nine *Ablp*^eyD^ strains, suggesting that the phenotype may not arise from the same causal mutation in each background. Given this absence of a shared candidate variant, we examined nearby regulatory features within the mapped interval. Within this interval, strain 662 carrying *Ablp*^*eyD*^ has an 8 kb *roo* element insertion within a predicted TFBS for *paired* (*prd*) (Supplemental Fig. S10; MacArthur et al. 2009), a key regulator of segmental patterning in *Drosophila* development (Kilchherr et al. 1986). This TFBS is located between *drumstick* (*drm*) and *sister of odd and bowl* (*sob*), both members of the odd-skipped gene family involved in leg joint formation (Hao et al. 2003). However, deletion of the TFBS (Δ886) using CRISPR-Cas9 produced no detectable leg joint phenotype (Supplemental Figs. S10, S11). In contrast, an independent 11 bp deletion (Δ11) resulted in abnormal development of the first tarsal joint, the same segment affected in the *Ablp*^eyD^ phenotype (Supplemental Figs. S12, S13). Examination of a publicly available ATAC-seq data (Huynh et al. 2023) suggests that the Δ11 deletion overlaps an accessible chromatin peak in the wing and eye discs but not in the brain or ovary, suggesting a stage- and tissue-specific regulatory role (Supplemental Fig. S10). Notably, the *roo* element itself is absent from the eight other stocks carrying the *Ablp*^eyD^ allele (Supplemental Table S4). Although the *roo* element could epigenetically silence the regulatory site (Lee and Karpen 2017), thereby causing the tarsal phenotype, a different mutation at this regulatory site may underlie the *Ablp*^eyD^ phenotype.

### Prevalence of SVs in deleterious phenotypes

**Figure 3.**
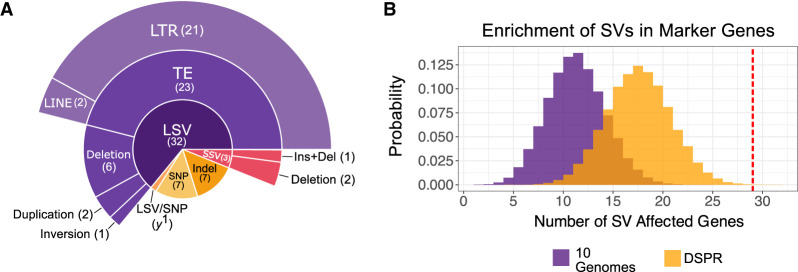
Enrichment of SVs among genes underlying visible phenotypes. (*A*) Distribution of candidate mutation classes identified across the 50 visible phenotypes analyzed in this study. Mutation classes include large structural variants (LSVs; >100 bp), small structural variants (SSVs; 11–100 bp), and transposable element (TE) insertions, with TEs further classified as long terminal repeat (LTR) or long interspersed nuclear element (LINE) retrotransposons. (*B*) Monte Carlo null distributions of the expected number of genes harboring at least one SV, generated by randomly sampling 43 genes matched for gene length from the genome-wide SV map across 10 assemblies (purple) and from the DSPR SV map (orange). The observed number of marker genes containing a candidate SV (29 of 43) is indicated by the red vertical line.

We found that 66% (33/50) of markers are associated with LSVs and 6% (3/50) with SSVs (Fig. 3A; Table 2). The remaining phenotypes are associated with SNPs or small indels that disrupt protein-coding sequences or regulatory sequences. Of the phenotypes in our data set caused by spontaneous or natural mutations, 71% (32/45) are associated with SVs. Similar to previous observations that TEs cause many visible phenotypic changes in *D. melanogaster* (Sankaranarayanan 1988), 46% (23/50) of mutations are associated with TEs. Notably, 21 of the 23 TE insertions associated with visible phenotypes in our data set are LTR retrotransposons, consistent with prior genomic analyses showing that LTR elements represent a major and functionally impactful class of TEs in *D. melanogaster* (Kaminker et al. 2002; Bergman et al. 2006). We also find duplication CNVs, indels, and inversions underlying the mutations (Table 2; Supplemental Table S7).

**Table 2.** Fifty visible phenotypes and candidate mutations

Mutation	Strain name(s)	Mutation type	Origin	Reference	Description
*ac* ^1^	6027	LSV/deletion	Spontaneous	Campuzano et al. 1985	2.8 kb deletion, located 5.8 kb upstream of *ac*
*Adc* ^b-1^	156	Indel	Spontaneous	Phillips et al. 2005	4 bp replaced by an 8 bp insertion in coding sequence; nonsynonymous SNP
*al* ^1^	156	Indel	Spontaneous	Uncharacterized	10 bp deletion of coding sequence
*ast* ^1^	1349	LSV/TE insertion	Spontaneous	Uncharacterized, but mutation type predicted by Higson et al. (1993)	Duplication of 6.4 kb region containing complete asteroid gene, copies separated by 33.8 kb insertion of *HMS-Beagle*, *gypsy*, and *roo* elements
*c* ^1^	156	LSV/TE insertion	Spontaneous	Uncharacterized	7.5 kb *DM412* element inserted in coding sequence
*ca* ^1^	576	SSV/deletion	Spontaneous	Ma et al. 2004	14 bp deletion of coding sequence
*car* ^1^	6027	SNP	X-ray	Sevrioukov et al. 1999	Nonsynonymous SNP
*cl* ^1^	1349	LSV/TE insertion	Spontaneous	Giordano et al. 2003	7.4 kb *blood* element inserted in coding sequence
*cm* ^1^	1282, 1570, 6027	LSV/TE insertion	Spontaneous	Mullins et al. 1999	9.2 kb *roo* element inserted in coding sequence
*cp* ^1^	620	SNP/indel	Spontaneous	Uncharacterized	Potentially a 7 bp indel near a regulatory site in *CG5151*
*ct* ^1^	6027	LSV/TE insertion	Spontaneous	Uncharacterized	7.3 kb *gypsy* element inserted in wing enhancer
*ct* ^6^	1570	LSV/TE insertion	Spontaneous	Tchurikov et al. 1989; Dorsett 1993	7.3 kb *gypsy* element inserted in wing enhancer
*cu* ^1^	576	Indel	Spontaneous	Grönke et al. 2009	Substitution of 2 bp at splice acceptor site
*Diap*1^th-1^	576	SNP/indel	Spontaneous	Uncharacterized	576 and 620 do not share any unique mutations in or near the gene; both have indels/SNPs in close proximity located in an intron, a putative enhancer region
	620				
*dpp* ^d-ho^	1349	LSV/deletion	Spontaneous	Masucci et al. 1990	3066 bp deletion, 22.7 kbp from 3′ end of *dpp*
*dpy* ^ov1^	156, 1349	LSV/TE insertion	Spontaneous	Carmon et al. 2010	7.4 kb *blood* element intronic insertion
*dy* ^1^	1570	LSV/deletion	spontaneous	DiBartolomeis et al. 2002	358 bp deletion, 1.8 kb from start of *dy*; 464 bp deletion, 2.7 kb from start of *dy*
*e* ^s^	554, 576	SNP	Spontaneous	Uncharacterized	Nonsynonymous SNP
*ed* ^1^	1349	LSV/TE insertion	Spontaneous	Uncharacterized	7.8 kb intronic insertion of *gypsy* and *Stalker* elements
*f* ^1^	1570	LSV/duplication	Spontaneous	New allele	2.7 kb duplication of coding exon and intronic sequence
	5295, 6027	LSV/TE insertion		Hoover et al. 1993	7.4 kb *gypsy* element intronic insertion
*g* ^1^	6027	LSV/TE insertion	Spontaneous	Uncharacterized, but mutation type predicted by Lloyd et al. (1999)	7.4 kb *blood* element intronic insertion
*g* ^2^	1570, 5295	SNP/indel	Spontaneous	Uncharacterized, but mutation type predicted by Lloyd et al. (1999)	3 bp in-frame deletion, nonsynonymous SNP
*hry* ^1^	576	LSV/TE insertion	Spontaneous	Hooper et al. 1989	7.4 kb *gypsy* element insertion, 4.8 kb from start of *hry*
*in* ^1^	620	SNP	Spontaneous	Uncharacterized	Nonsynonymous SNP
*kni* ^ri-1^	620	LSV/deletion	Spontaneous	Lunde et al. 2003	253 bp deletion of regulatory sequence, 11.7 kb from start of *kni*
*m* ^1^	6027	SSV/indel	Spontaneous	Roch et al. 2003	11 bp deletion of coding sequence
*m* ^74f^	1282	Indel	Ethyl methanesulfonate	Uncharacterized	1 bp deletion, 3 bp intronic insertion
*oc* ^1^	1570	LSV/inversion	X-ray	Spradling et al. 1979; Spradling and Mahowald 1981	154 kb inversion, including *oc* gene
*p* ^p^	620	SNP	Spontaneous	Falcón-Pérez et al. 2007; Syrzycka et al. 2007)	Nonsynonymous SNP
*pn* ^1^	1570, 6027	LSV/TE insertion	Spontaneous	Frolov et al. 1994; Timmons and Shearn 1996	7.5 kb *DM412* element inserted in coding exon
*pr* ^1^	156	LSV/TE insertion	Spontaneous	Kim et al. 1996	7.5 kb *DM412* element intronic insertion
*px* ^1^	156	LSV/Duplication	Spontaneous	Uncharacterized	Partial duplication of coding exon with copies separated by 7.4 kb *DM412* element
*ras* ^2^	1570	LSV/TE insertion	Spontaneous	Location and size approximated by Nash et al. (1994)	5 kb *blastopia* intronic insertion
*ras* ^4^	6027	LSV/TE insertion	Spontaneous	Uncharacterized, might be misgenotyped, same mutation as ras^2^	5 kb *blastopia* intronic insertion
*rb* ^1^	6027	LSV/TE insertion	Spontaneous	Uncharacterized	7.8 kb insertion of *gypsy* and *Stalker* elements into coding exon
*ru* ^1^	576	SNP	Spontaneous	Yu et al. 2010	Premature stop codon
*sc* ^1^	6027	LSV/TE insertion	Spontaneous	Campuzano et al. 1985	1.6 kb *gypsy*, 7 kb from start of *sc*
*sd* ^1^	1282	SSV/deletion	X-ray	Uncharacterized	22 bp deletion of coding sequence
*sn* ^3^	5295, 6027	LSV/deletion	Spontaneous	Paterson and O'Hare 1991	292 bp deletion from 5′ UTR
*speck* ^1^	156	LSV/TE insertion	Spontaneous	Chakraborty et al. 2019; Spana et al. 2020	7.5 kb *DM412* element inserted into intron, present in ISO-1 reference
*sr* ^1^	554, 576	SNP	Spontaneous	Uncharacterized	576 and 554 share four SNPs in 5′ UTR and three intronic SNPs
*st* ^1^	576, 620	LSV/TE insertion	Spontaneous	ten Have et al. 1995	7.4 kb *DM412* element inserted in coding exon
*sv* ^de^	662	LSV/TE insertion	Spontaneous	Fu et al. 1998	5 kb Tirant 1.6 Kb from 5′ end of gene
*Ubx* ^bx-1^	554	LSV/TE insertion	Spontaneous	Bender et al. 1983; Castelli-Gair and García-Bellido 1990	7.5 kb *DM412* element insertion into intronic regulatory element
Df(1)os-o(*upd*1^os-o^,*upd*3^os-o^)	1570	LSV/deletion	X-ray	Wang et al. 2014	3.1 kb deletion upstream of upd^3^
Ab(1)os-s(*upd1*^os-s^,*upd3*^os-s^)	1282	SNP/Indel	Spontaneous	Uncharacterized	Nonsynonymous SNP and 3 bp in-frame deletion of coding sequence in *upd*^3^
*v* ^1^	1570, 5295, 6027	LSV/TE insertion	Spontaneous	Searles et al. 1990	7.5 kb *DM412* element inserted in 5′ UTR
*w* ^1^	1570, 6027	LSV/TE insertion	Spontaneous	Driver et al. 1989	4.7 kb *Doc* element insertion, 17 bp from start of *w*
*w* ^1118^	5295	LSV/TE insertion	Spontaneous	Hazelrigg et al. 1984	4.7 kb *Doc* element insertion, 4 kb *copia* within *Doc* element, 17 bp from start of *w*
*y* ^1^	6027	LSV/insertion	Spontaneous	New allele	622 bp insertion in coding exon, introduces premature stop codon
	1570, 5295	SNP		Geyer et al. 1990	Start codon loss caused by SNP

To determine whether SVs are associated with visible phenotypic changes disproportionately compared with the average SV burden in euchromatic genes, we compared the prevalence of SVs in the 43 genes linked to the 50 phenotypic markers with their genome-wide distribution across the 10 genome assemblies in which these mutations were identified. We excluded strain 2969, which was included in this study to assess assembly performance on the *Bar* tandem duplication and carries no additional marker mutations. We performed a Monte Carlo simulation, randomly drawing 100,000 gene sets matched for gene length to our marker gene set to generate a null distribution for the expected number of SVs (Methods). Based on this null model, we would expect SVs in 12 genes by chance; however, we observed SVs in 29 of the 43 marker genes, representing an enrichment of 141.67% (*P*-value = 9.99 × 10^−6^) (Fig. 3B). Because the analyzed genomes were generated by crossing multiple strains to combine marker mutations, their SV content may differ from the spectra of mutations in chromosomes segregating in natural populations. To address this, we repeated the analysis using SV calls from the genome assemblies of 14 inbred strains collected from various geographical locations worldwide (Chakraborty et al. 2019). Given the abundance of SVs in this population sample, we expect to observe SVs in 18 genes by chance. The observation of 29 genes with SVs in the marker set thus represents a 61.1% enrichment (*P*-value = 7.90 × 10^−4^) (Fig. 3B). To test whether this enrichment reflects a generally elevated mutation rate in these genes, we performed analogous length-matched enrichment analyses for nsSNPs and small indels. In contrast to SVs, marker genes show no enrichment for either mutation type, indicating that the excess of SVs is not attributable to broadly hypermutable or fragile genomic regions (Supplemental Fig. S14).

All 50 phenotypes examined in this study are associated with visible phenotypic changes, and 44 among these have deleterious effects on health and behavior (Supplemental Table S8). For instance, the vermilion eye color mutation (*v*^1^) causes slow and irregular heart rates (Beasley and Dowse 2016); *white* eye color mutations (*w*^1118^ and *w*^1^) are linked to defects affecting mobility, life span, and courtship behavior (Krstic et al. 2013; Xiao et al. 2017; Arimoto et al. 2020); Bristle mutants (*forked*) exhibit a reduced response to courtship sounds (Cosetti et al. 2008); and yellow body color mutant (*yellow*) males have lower mating success owing to reduced melanization of their sex combs (Massey et al. 2019). Among the 44 markers associated with deleterious fitness effects, 75% (33/44) are associated with an SV.

### Allelic diversity underlying phenotypic changes

Similar phenotypes can often result from distinct mutations in the same gene (Schmidt et al. 2010; King et al. 2014; Chakraborty et al. 2019; GTEx Consortium 2020), although the prevalence of multiple alleles at loci underlying variation in deleterious organismal phenotypes remains unclear. To examine the diversity of molecular alleles underlying the deleterious phenotypes in our data set, we inspected the genes underlying the 12 phenotypes present in more than one strain. We identified multiple SV alleles linked to four phenotypes shared between strains, including three previously unknown.

**Figure 4.**
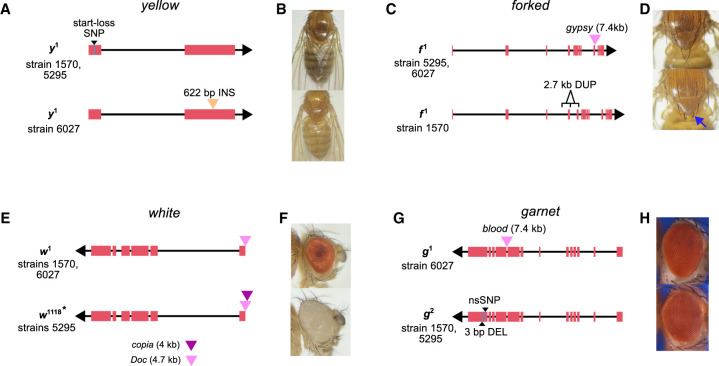
Molecular diversity underlying classical visible phenotypes. (*A*) Two strains exhibiting the yellow body color phenotype carry the previously characterized SNP that disrupts the start codon. In contrast, strain 6027 harbors a 622 bp insertion in the second exon, resulting in a frameshift and a premature stop codon. (*B*) Phenotype images of wild-type (*top*) and *y*¹ mutant (*bottom*) body color. (*C*) The *forked* (*f*¹) allele was previously associated with a TE insertion; however, strain 1570 lacks this insertion and instead carries a 2.7 kb tandem duplication encompassing a complete exon. (*D*) Phenotype images of wild-type (*top*) and *f*¹ mutant forked bristles (*bottom*, blue arrow). (*E*) Two alleles of the *white* gene are associated with TE insertions near the transcription start site. A 4.7 kb *Doc* element insertion is present in strains 1570 and 6027, and a nested *copia* element within the *Doc* insertion is observed in strain 5295, consistent with recurrent insertions at the same genomic site. (*F*) Phenotype images of wild-type (*top*) and *white* mutant (*bottom*) eye color. (*G*) Two alleles of *garnet* involve either an intronic *blood* element insertion (*g*¹) or a nonsynonymous SNP with an in-frame deletion in the final coding exon (*g*²). (*H*) Phenotype images of wild-type (*top*) and *g*¹ mutant eye color, obtained from FlyBase (FBrf0220532).

Notably, two phenotypes are classic, well-characterized mutations: *yellow*, which results in a yellow body color, and *forked,* which results in forked bristles. Our analysis revealed previously uncharacterized molecular diversity underlying these phenotypes. For example, strains 1570, 5295, and 6027 possess the *y*^1^ mutation, although only strains 1570 and 5295 carry the previously characterized start-codon-loss mutation caused by an SNP in the initiation codon of the *y* gene (Geyer et al. 1990). The strain 6027 instead has a 622 bp insertion in a protein-coding exon of *y* (Fig. 4A,B). This insertion disrupts the ORF and introduces a premature stop codon, likely leading to a truncated *y* protein. Similarly, *f*^1^ is thought to be caused by an intronic *gypsy* TE insertion (Hoover et al. 1993). Although strains 1570, 5295, and 6027 show the forked mutant phenotype, only strains 5295 and 6027 have the TE. The *f* gene in 1570 harbors a tandem duplication that copies exonic and intronic sequences, likely disrupting the ORF and producing a mutant phenotype (Fig. 4C,D).

We also identified a new molecular basis for mutations documented as alleles of the same gene. For instance, *w*^1^ and *w*^1118^ are alleles of the *white* gene, both of which are associated with loss of eye pigmentation. The *w*^1^ allele was previously linked to a TE insertion near the transcription start site, and we confirm this by finding a 4.7 kb *Doc* insertion in strains 1570 and 6027 (Fig. 4E,F). Although the strain 5295 was originally labeled as *w*^1118^, our analysis revealed that it lacks the canonical deletion previously associated with this allele (Hazelrigg et al. 1984). Instead, comparison to additional *w*^1118^ assemblies indicates that the allele in strain 5295 more closely resembles *w*^1^, with an additional nested 3.5 kb *copia* element inserted within the *Doc* element (Fig. 4E). These insertions occur at the same genomic site, suggesting that recurrent TE insertions at this locus may give rise to the *white* eye phenotype. We also find evidence that distinct mutation types can have similar phenotypic effects. *g*^1^ and *g*^2^ are alleles of the eye color gene *garnet* (*g*). We show that *g*^1^ is associated with an intronic 7.4 kb *blood* insertion, whereas *g*^2^ is linked to an in-frame 3 bp deletion and an nsSNP in the last exon (Fig. 4G,H).

Additionally, two sets of mutations listed as distinct alleles in the stock genotypes may share the same molecular basis. *ct*^1^ and *ct*^6^ are both alleles of the *cut* gene and are associated with wing notches. The molecular basis of *ct*^6^ has been characterized and is caused by a *gypsy* TE inserted between the *cut* promoter and a distant wing-margin enhancer (Dorsett 1993; Cai and Levine 1997). We found that strains 6027 and 1570, which are genotyped as *ct*^1^ and *ct*^6^, respectively, both carry this same *gypsy* TE insertion at the same position, with no other unique disruptive mutations in or near the *cut* gene. Likewise, *ras*^2^ and *ras*^4^ are alleles of the eye color gene *raspberry*. *ras*^2^ is linked to a 5 kb *blastopia* insertion, and both strains 1570 and 6027 carry this same TE insertion at the same site, again with no other clear disruptions in the gene. These findings suggest that recorded genotypes for these stocks may be incorrect or that the alleles may share the same underlying mutation. The presence of phenotypic differences despite identical disruptions could also mean that additional genetic modifiers, located outside of the previously mapped gene regions, may influence the observed phenotypes. Variation in piRNA-mediated silencing across genetic backgrounds may also modulate TE activity or nearby gene expression, providing another potential source of phenotypic divergence (Kelleher et al. 2012; Sentmanat and Elgin 2012).

## Discussion

SVs underlie both deleterious and adaptive phenotypic changes and have been hypothesized to account for a portion of the missing causal variants in complex trait variation (Manolio et al. 2009; Eichler et al. 2010). However, their overall impact on phenotypic variation, including fitness-related traits, remains poorly defined. In *D. melanogaster,* early studies showed that many spontaneous visible mutations involve TEs (Green 1988; Sankaranarayanan 1988), but these analyses focused on phenotypes already known to be associated with TEs. As a result, the broader contribution of SVs—both TE and non-TE—has remained unclear. By constructing a comprehensive variant map across 11 strains carrying 50 classical visible mutations, we show that SVs are associated with a disproportionately large fraction of phenotypic changes compared with SNPs, including eight previously undetected SVs. These findings mirror growing evidence from model organisms (Chakraborty et al. 2019), agriculture (Chia et al. 2012; Alonge et al. 2020), livestock (Li et al. 2024; Yang et al. 2024), and humans (Abel et al. 2020), indicating that hidden SVs likely underlie a wide range of traits, from physiology to behavior.

Although the variants underlying these visible phenotypes were not sampled from natural populations, their nature—often single, large-effect mutations—is similar to phenotypic differences observed in diverse biological contexts, including morphological and behavioral divergence across taxa and domestication traits in crops and livestock (Naisbit et al. 2003; Shapiro et al. 2004; Hoekstra et al. 2006; Andersson 2013; Wills et al. 2013). Our data set consists mostly of spontaneous mutations identified in laboratory stocks; however, these mutations serve as useful models for understanding how large mutations contribute to phenotypic variation. Because purifying selection is weakened in laboratory conditions, owing to both controlled environments and small effective population sizes, deleterious mutations can persist longer in laboratory stocks than in natural populations. This context helps explain why these large-effect alleles remain observable in laboratory strains despite their likely negative fitness effects in wild populations.

Consistent with population genetic analyses showing that SVs, particularly TEs and duplications, segregate at low frequencies (Chakraborty et al. 2019; Collins and Talkowski 2025), we infer that 44 of the phenotypes examined in this study are deleterious, with the majority (75%) associated with SVs. Although our conclusion of enrichment refers specifically to this historically unbiased set of classical visible phenotypes, and not to all trait categories genome-wide, deleterious recessive mutations, such as these marker phenotypes, often segregate at low frequencies in natural populations. Because the shape of the allele-frequency distribution of SVs in our strains closely resembles that observed in natural populations (Chakraborty et al. 2019), the enrichment of SVs among deleterious phenotypes could provide a biological basis for the population genetic inferences that SVs often exert stronger harmful effects than nonsynonymous SNPs. Additionally, our data show that similar deleterious phenotypic changes can result from different SV alleles, with the proportion of phenotypes (3/12) exhibiting allelic heterogeneity comparable to that of *D. melanogaster* genes (3/12). The prominent role of hidden and multiallelic SVs in deleterious phenotypes highlights their potential contribution to disease genetics and unexplained heritability in complex traits (Manolio et al. 2009; Eichler et al. 2010), as well as to inbreeding depression and extinction risk in small populations experiencing weak natural selection (Rogers and Slatkin 2017).

SVs can influence gene structure and function through diverse mechanisms. Although duplications of complete genes have long been recognized as drivers of phenotypic variation and adaptation (Hughes 1994; Chakraborty and Fry 2015; Cardoso-Moreira et al. 2016), genomic rearrangements involving partial genes can also have profound effects. Classic examples include the origin of the *jingwei* gene in *Drosophila* (Long and Langley 1993) and disease-causing exon duplications in humans (White et al. 2006). Consistent with these patterns, we show that partial gene duplications and noncoding SVs—including variants affecting UTRs, introns, and intergenic regions—account for more than one-half (27/50) of the candidate mutations identified here, reinforcing the importance of noncoding variation in shaping phenotypes (Maurano et al. 2012; Alsheikh et al. 2022; Schipper and Posthuma 2022).

Although reference-based methods are widely used, they often fail to detect complex or large SVs (Chakraborty et al. 2018). In contrast, de novo assemblies and pangenome approaches can capture the full size and structure of complex variants, as illustrated by tandem duplications and TE-associated rearrangements such as *plexus* and *asteroid*. Together, these examples show that assembly- and pangenome-based approaches can reveal functionally important SVs, including novel molecular alleles in highly studied genes, that would be hidden with read mapping alone and thereby lead to misleading inferences about the mutational basis of phenotypic variation.

Beyond variant discovery, long-read assemblies enable improved genome annotation. *D. melanogaster*, despite being one of the best-annotated metazoan genomes, still contains genes that have not been mapped to the genome sequence (Dean et al. 2022). We examined three such cases—*curved*, *clipped*, and *Ablp—*and identified genes and a putative regulatory element likely responsible for the associated mutant phenotypes. These results highlight how modern genomic approaches can close persistent gaps in the genotype–phenotype map, even in *D. melanogaster*. Inspired by A.H. Sturtevant's pioneering undergraduate work in genetic mapping, we integrated this model in a resource called genomics and long reads education (GALORE) that embodies the same spirit of discovery in the modern classroom, while also supporting more advanced training and research applications, such as recombination landscape inference (see Data access).

The discovery of the *Bar*^1^ mutation in *Drosophila* provided the earliest evidence supporting the role of genome structural changes in phenotypic variation (Hurles et al. 2008). Since then, SVs have been implicated in several Mendelian and complex diseases as well as adaptations (Merker et al. 2018; Quan et al. 2021; Collins and Talkowski 2025). However, the extent to which SVs contribute to phenotypic variation remains unclear, partly because of the challenges of detecting comprehensive SVs. Comparative genomics using highly contiguous genome assemblies have largely solved that problem, although our understanding of the contribution of SVs in phenotypic variation remains incomplete. Consistent with previous findings of enrichment of SVs in candidate genes in QTL mapping experiments, our results suggest a disproportionate role of SVs in spontaneous, deleterious changes in phenotypes with both Mendelian and complex genetic bases (Chakraborty et al. 2019). Thus, our results further show that SVs can act as rare, large-effect alleles and may account for undetected causal mutations underlying variation in Mendelian and complex traits, particularly those affecting organismal fitness.

## Methods

### Fly stocks and DNA extraction

We obtained the *D. melanogaster* stocks from the Bloomington Stock Center (ordered on December 10, 2023; received on December 18, 2023). The presence of visual markers listed on the stock center website was verified for each strain. We collected 150 females from each stock and extracted high-molecular-weight DNA using the method described by Chakraborty et al. (2016). Briefly, we flash-froze the flies in liquid nitrogen and ground them into fine powder using a mortar and pestle. We extracted DNA from the fly powder using the Qiagen Blood and Cell Culture DNA Midi Kit and then spooled the DNA at the final stage using a glass hook.

### Library preparation and sequencing

We prepared the ONT library for each strain following the manufacturer's ligation kit protocol. The initial concentration and total volume of DNA for each sample are provided in Supplemental Table S2. For high-molecular-weight DNA from strains 2969, 5295, 576, and 1349, we used the Pacific Biosciences (PacBio) short-read eliminator XL kit to remove DNA fragments <40 kb in length. DNA was end-repaired using the NEBNext companion module for ONT ligation sequencing kit (New England Biolabs), followed by adaptor ligation with the ONT duplex-enabled ligation sequencing kit v14. Libraries were sequenced on R10.4.1 flow cells using a MinION Mk1B for 72 h.

### Base calling and assembly

We performed base calling using a Dorado duplex base calling model on a laptop with 64 GB of memory and 2 TB of SSD storage. Although each run produced a small proportion of duplex reads, we did not assemble them separately because of their low coverage (Supplemental Table S3). Raw ONT reads were filtered using Chopper (De Coster and Rademakers 2023), keeping only reads with an average Phred quality score greater than 10 and lengths greater than 10 kbp. We generated draft assemblies for each genome using Hifiasm v0.25.0 (Cheng et al. 2021). Microbial contigs in the draft assembly were identified using BlobTools v1.1 (Laetsch and Blaxter 2017). For input to the BlobTools analysis, we generated a taxonomic annotation file by aligning contigs against the NCBI Nucleotide database (https://www.ncbi.nlm.nih.gov/nucleotide/) (downloaded on April 23, 2024) using BLASTN. The read-mapping input was generated by mapping the quality-filtered ONT reads to the draft assembly using minimap2 v2.26 (Li 2017). Only contigs classified as “Arthropoda” or “no-hit” were retained. Cleaned, draft assemblies were polished using Medaka (https://github.com/nanoporetech/medaka). Polished contigs were scaffolded using *mscaffolder* (Chakraborty et al. 2018) with release 6.49 of the ISO-1 genome assembly (Hoskins et al. 2015) as the reference.

Strain 662 carries the *ey*^*D*^ mutation, which involves a translocation duplication of a segment from Chromosome 2L onto the fourth chromosome. Hifiasm was unable to resolve this complex SV, so we used Flye v2.9.3 to generate and inspect the repeat graph (Kolmogorov et al. 2019). The repeat graph was visualized using Bandage (Wick et al. 2015), which indicated that the duplicated sequence may have been collapsed into a single copy (Supplemental Fig. S5). To recover the entire structure, we manually expanded the collapsed region by exporting the path as a FASTA sequence. We confirmed the new breakpoints by mapping reads back to the duplicated sequence (Supplemental Fig. S5).

### Assembly quality assessment

To evaluate assembly quality, we first identified potential large-scale misassemblies by remapping long reads to the contig assemblies using minimap2 v2.26 (Li 2017) and visually inspecting regions with abnormal coverage profiles. Assembly contiguity was quantified using QUAST v5.0.2 (Gurevich et al. 2013), based on standard summary statistics (contig N50, contig number, total assembly size). Completeness was assessed using BUSCO v5.7.1 (Simão et al. 2015) with the Diptera ortholog database downloaded on July 8, 2024 (https://busco-data.ezlab.org/v5/data/lineages/diptera_odb10.2024-01-08.tar.gz).

Assembly consensus accuracy (QV) was estimated by mapping raw ONT reads back to each assembly and calling variants with PEPPER-Margin-DeepVariant using the model trained on ONT R10 sequencing data (Shafin et al. 2021). We treated homozygous nonreference (1/1) variants with filter=pass and genotype quality > 10 as high-confidence discrepancies between the reads and the assembly. The number of such sites was divided by the assembly size (in base pairs) to obtain an estimated error rate, which was converted to a Phred-scaled quality value:

$$\mathrm{QV}=-10\log_{10}(\mathrm{number}\;\mathrm{of}\;\mathrm{errors}/\mathrm{assembly}\;\mathrm{size}).$$

This ONT-only, read-mapping-based approach is consistent with prior methods used to assess consensus accuracy in long-read genome assemblies when Illumina data are unavailable (Koren et al. 2017; Solares et al. 2018).

### Repeat annotation and SV calling

We annotated repeats in each assembly using RepeatMasker v4.1.2 (Smit et al. 2013). To identify SVs, we combined whole-genome alignment and read-mapping approaches. We utilized the Minigraph-Cactus pipeline (Hickey et al. 2023) to construct a pangenome graph, encompassing all forms of genetic variation across the 11 genomes. From the ISO1-based VCF file, we identified mutations located within annotated genes that were unique to strains carrying marker mutations. Additionally, each assembly was aligned to the ISO-1 reference genome, specifically the major chromosome arms and the dot chromosome (X, 2L, 2R, 3L, 3R, 4), using MUMmer v4 (Marçais et al. 2018). Structural differences between assemblies were then classified as insertions, deletions, duplications, or inversions using SVMU (Chakraborty et al. 2019). Finally, we mapped the ONT reads to the ISO-1 reference using minimap2 v2.26 (Li 2017) and inspected them in the Integrative Genomics Viewer (IGV) (Thorvaldsdóttir et al. 2013) to confirm that the read data supported SVs detected by whole-genome alignment-based methods. Strains 1570 and 6027 carry an X Chromosome balancer, so the assembled X Chromosomes are highly fragmented. To identify mutations linked to X-Chromosome marker genes in these strains, we used Sniffles v2.6.1 (Smolka et al. 2024) to detect SVs from reads mapped to the ISO-1 reference. Candidate SVs were similarly verified in IGV.

### SNP and indel calling

SNPs and indels were called using PEPPER-Margin-DeepVariant (Shafin et al. 2021), which uses models trained on ONT R10 data and provides higher base-level accuracy than that of graph-derived calls from Minigraph-Cactus. A comparison of small variant calls (Supplemental Fig. S15) demonstrated that although both tools share a high concordance of variants, PMDV captures a large number of high-quality SNPs and small indels, particularly heterozygous sites, that are absent or collapsed in the pangenome graph. Only PMDV variant calls with a “pass” filter were included in downstream analyses. Individual strain VCF files were normalized and merged with BCFtools v1.19 (Danecek et al. 2021), and variant effects were predicted using SnpEff v5.2 (Cingolani et al. 2012).

### CRISPR genome editing

To examine the functional significance of the predicted regulatory region for the unmapped *Ablp* gene, we selected two guide RNAs (gRNA1: 5′-TGATTGCGAAGAAACCTCTG-3′; gRNA2: 5′-TTGACAGGCAACTGGCGATC-3′) flanking the predicted enhancer site using the CRISPOR web tool (Concordet and Haeussler 2018). gRNA2 is positioned 84 bp downstream from the *roo* insertion site and near the 3′ end of the predicted *paired* binding site; gRNA1 targets the opposite flank, 806 bp from the *roo* insertion site, to enable full-site deletion. As the gRNA2 sequence is located very close to the 3′ end of the predicted *Paired* binding site, deletions resulting from CRISPR-Cas9-mediated DNA breaks may overlap and disrupt the regulatory site. The synthesized gRNAs (Synthego) were incubated with the Cas9 enzyme (Synthego) to form RNPs and then injected into embryos of the BDSC strain 54591 (Port et al. 2014). The presence of the gRNA sequence was verified by aligning the putative regulatory sequence from the ISO-1 reference sequence to the genome sequence of strain 54591 available at GitHub (https://github.com/chakrabortymlab/DLPD). Embryo injections were performed by GenetiVision. Surviving females were individually crossed to single males and allowed to mate for 5 days. Following the observation of larval activity, females (G_0_) were genotyped by PCR and amplicon sequencing to assess CRISPR-mediated deletions.

To genotype individuals for CRISPR-mediated deletion alleles, we isolated genomic DNA from individual flies using the Monarch genomic DNA purification kit (New England Biolabs) according to the manufacturer's genomic DNA extraction from insects protocol. The genomic DNA was amplified using Q5 polymerase with the primers 5′-TCAGCGAGTACAACTCAGCA-3′ and 5′-TTGTTGTCGCTGGAGATTCGA-3′. The PCR amplicons were purified using the Monarch PCR & DNA cleanup kit and sequenced with Oxford Nanopore (Plasmidsaurus). The sequencing reads were mapped to the 54591 assembly using minimap2 v2.26 (Li 2017). The read alignments in BAM format were viewed in IGV to examine the CRISPR-induced deletions. We recovered two deletion alleles: a full deletion between the two gRNAs and an 11 bp deletion at the site of the second gRNA. The presence of only reads carrying a single deletion allele was considered as evidence for homozygosity of the deletion, whereas the presence of different edited alleles or a mix of edited and unedited alleles was considered as as evidence of heterozygous mutations.

F_1_ males and females from G_0_ females homozygous for the deletion were crossed and genotyped. Because of the unknown genotype of the G_0_ male, not all F_1_ males and females were homozygous for the deletion. Thus, we isolated F_2_ males and females homozygous for the deletion and inspected their leg phenotypes.

### Deletion mapping of *clipped* gene

Strain 620 carries a wing mutation, *cp*^1^, a mutant allele of the *clipped* gene, whose precise genomic location has not yet been determined. According to unpublished data on FlyBase (https://flybase.org/reports/FBrf0198635), *clipped* was predicted to reside within a 3.12 Mb region between the genes *asf1* and *st*. To refine the location of the *clipped* gene, we performed deletion mapping using 14 lines, each carrying a chromosomal deletion spanning different portions of the predicted interval (Supplemental Table S5). These deletion stocks were crossed to strain 620 as well as to strain 466, which also carries the *cp*^1^ allele, and the F_1_s were examined for the wing notching phenotype associated with *cp*^1^.

Eleven deletions complemented the *cp*^1^ phenotype, indicating that the mutation lies outside the regions deleted in those lines. Three deletions failed to complement, suggesting that these *overlapping* deletions uncover the *cp*^1^ mutation. From the overlap of the three noncomplementing deletions, we defined a minimal candidate interval of 54,992 bp on Chromosome 3L (Supplemental Fig. S7).

### Enrichment of SVs in mapped genes

To determine whether the number of marker genes with candidate SVs was significantly higher than the genome-wide distribution of SVs, we generated a null distribution from our SV map across 10 genomes. We excluded SVs from strain 2969 from this analysis, as we did not include the *Bar*^1^ mutation among our 50 markers because it is known to be an SV. We considered SVs >100 bp and identified euchromatic protein-coding genes located within 1000 bp of any SV. To construct a null distribution, we randomly sampled 43 genes (the number of genes linked to the 50 phenotypes) from the genome, controlling for gene length by matching the distribution of gene lengths in the marker gene set to those in the sampled sets using a kernel density estimation (KDE) and rejection sampling approach. This sampling procedure was repeated 100,000 times. The *P*-value was calculated using the formula *P* = (*r* + 1)/(*n* + 1) (North et al. 2002), where *n* is the total number of replicates, and *r* is the number of replicates in which the number of genes affected by SVs is equal to or larger than the observed number of marker genes with candidate SVs. This analysis was repeated with a map of euchromatic SVs in the *Drosophila* Synthetic Population Resource (DSPR), a panel of 14 isogenic lines derived from globally diverse populations (Chakraborty et al. 2019). We also performed an analogous enrichment analysis for nsSNPs and small indels (2–10 bp).

### Additional sequencing and analysis of newly characterized mutations

To further investigate candidate causal mutations underlying the *curved*, *clipped*, and *Ablp*^eyD^ phenotypes, we obtained independent strains carrying these mutations from the Bloomington Drosophila Stock Center (eight *Ablp*^eyD^, six *c*^1^, and six *cp*^1^ strains). We verified that all strains carried their associated phenotypes. High-molecular-weight DNA was extracted from all the strains and end-repaired as described above. Barcoded sequencing libraries were prepared using the Oxford Nanopore native barcoding kit 24 (v14) and sequenced on a PromethION R10.4.1 flow cell for 72 h.

SVs were genotyped by mapping reads from each strain to the pangenome graph using the vg toolkit, v1.65.0 (Garrison et al. 2018). Long reads were aligned with vg giraffe using an index optimized for Oxford Nanopore R10 data, producing GAF-format alignments. These alignments were converted into a packed coverage representation using vg pack. Variants were then genotyped directly from the pangenome graph using vg call, generating VCF files for each strain. SNPs and indels were genotyped using PEPPER-Margin-DeepVariant (Shafin et al. 2021). Individual strain VCF files were normalized and merged with BCFtools v1.19 (Danecek et al. 2021). A custom Python script, *merged_vcf_strain_filter.py*, was used to filter the merged VCF file to retain variants present in a specified group of strains and absent from all other samples. Candidate genotypes were further validated by mapping reads to the ISO-1 reference genome using minimap2 v2.26 (Li 2017), followed by visual inspection in IGV.

### ATAC-seq peak identification

To define regions of accessible chromatin, we used publicly available ATAC-seq data from *D. melanogaster* wing disc, eye disc, brain, and ovary (Huynh et al. 2023). Reads were trimmed with Trimmomatic v0.39 (Bolger et al. 2014) and aligned to the ISO-1 r6 reference genome using BWA-mem2 v2.2.1 (Vasimuddin et al. 2019). PCR duplicates were removed with Picard MarkDuplicates (http://broadinstitute.github.io/picard), and chromatin accessibility peaks were called using MACS2 v2.2.9.1 (Zhang et al. 2008). To identify candidate regulatory mutations underlying the *clipped* phenotype, we intersected variants unique to the seven *cp*¹ strains within the mapped 55 kb interval with ATAC-seq peaks and visually inspected peak support in IGV (Thorvaldsdóttir et al. 2013). Similarly, the functional relevance of CRISPR-induced deletions at the *Ablp*^eyD^ locus was evaluated based on their overlap with tissue-specific accessibility peaks.

### Direct RNA sequencing

Total RNA was extracted from 7-day-old male flies of strain 156 using TRIzol. RNA was reverse-transcribed to produce the DNA–RNA hybrids using the Induro reverse transcriptase kit, and sequencing libraries were prepared with the Oxford Nanopore direct RNA sequencing kit. Libraries were sequenced on a PromethION RNA flow cell for 72 h. Reads were mapped to both the ISO-1 reference genome and our de novo assembly for strain 156 using minimap2 v2.26 with splice-aware alignment parameters optimized for Oxford Nanopore RNA sequencing (-ax splice -uf -k14) (Li 2017). Gene annotations for *Strn-Mlck* and *plexus* were lifted over to the 156 assembly using LiftOff v1.6.3 (Shumate and Salzberg 2021).

## Data access

All sequencing reads and genome assemblies generated in this study have been submitted to the NCBI BioProject database (https://www.ncbi.nlm.nih.gov/bioproject/) under accession number PRJNA1214913. All scripts for genome assembly and analysis are available as Supplemental Code and at GitHub (https://github.com/chakrabortymlab/visible_phenotype). Additional undergraduate-level, step-by-step instructions to carry out the genomic analyses reported here are available at GitHub (https://github.com/chakrabortymlab/GALORE).

## Supplemental Material

Supplement 1

Supplement 2

Supplement 3

Supplement 4
