# Supplementary material for: Structural variants are enriched in deleterious visible phenotypes in Drosophila
Source: Genome Res. 2026 Jul;36(7):1369–85. doi: 10.1101/gr.281434.125 (PMC13322080; doi:10.1101/gr.281434.125)
Supplement: Supplement 1 [file Supplemental_Materials.pdf]

Supplemental Materials

Alejandra Samano, Matthew Musat, Mihir Junaghare, Asad Ahmad, Mehlum Ali, Sebastian Alves, Sreeram Pasupuleti, Jelisha Perera, Omar Saada, Brady Sabido, Trevor Smith, Sophie Walz, Mahul Chakraborty

Affiliations:
Department of Biology, Texas A&M University, College Station, TX 77843

Supplementary Figures

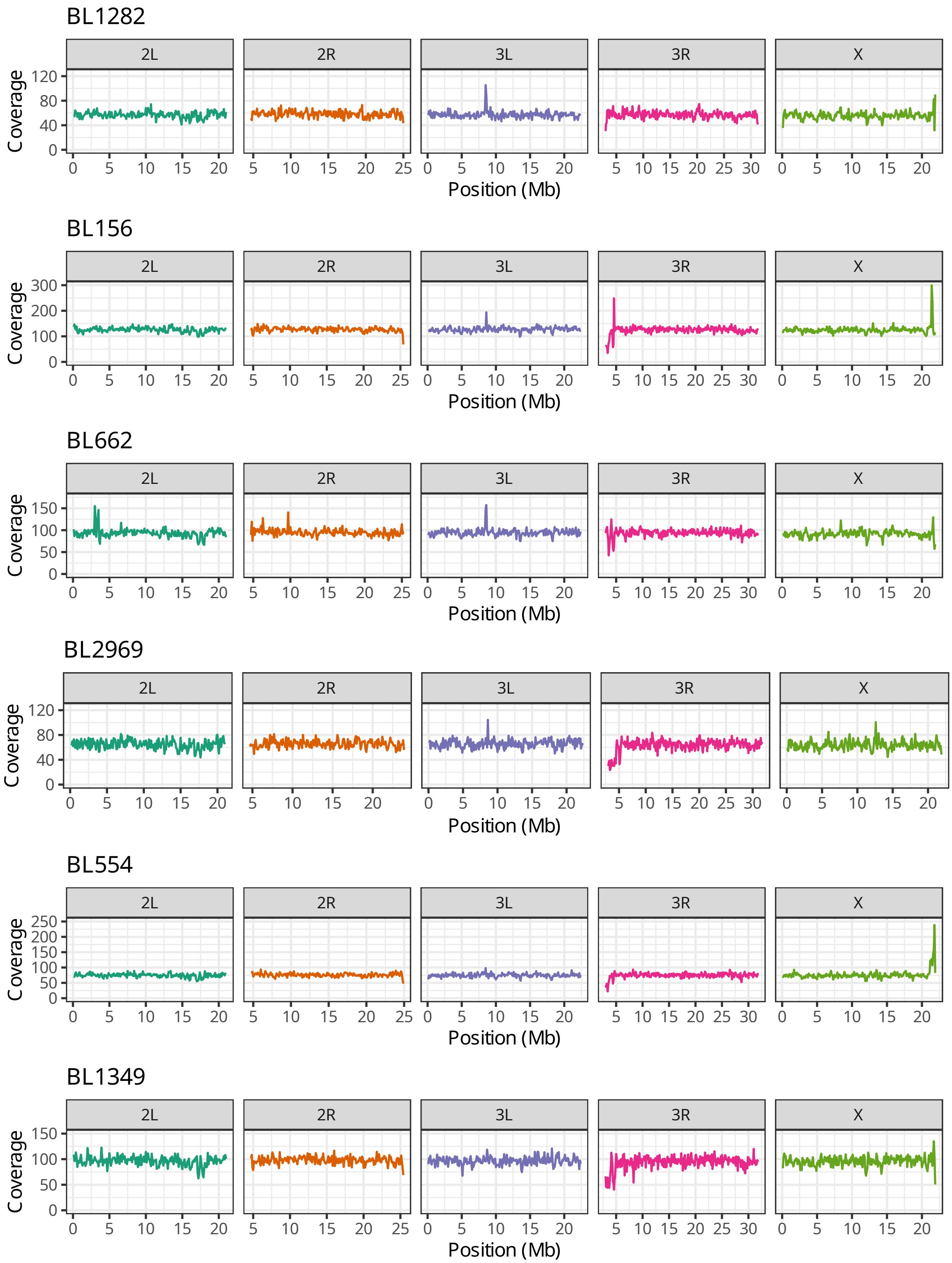

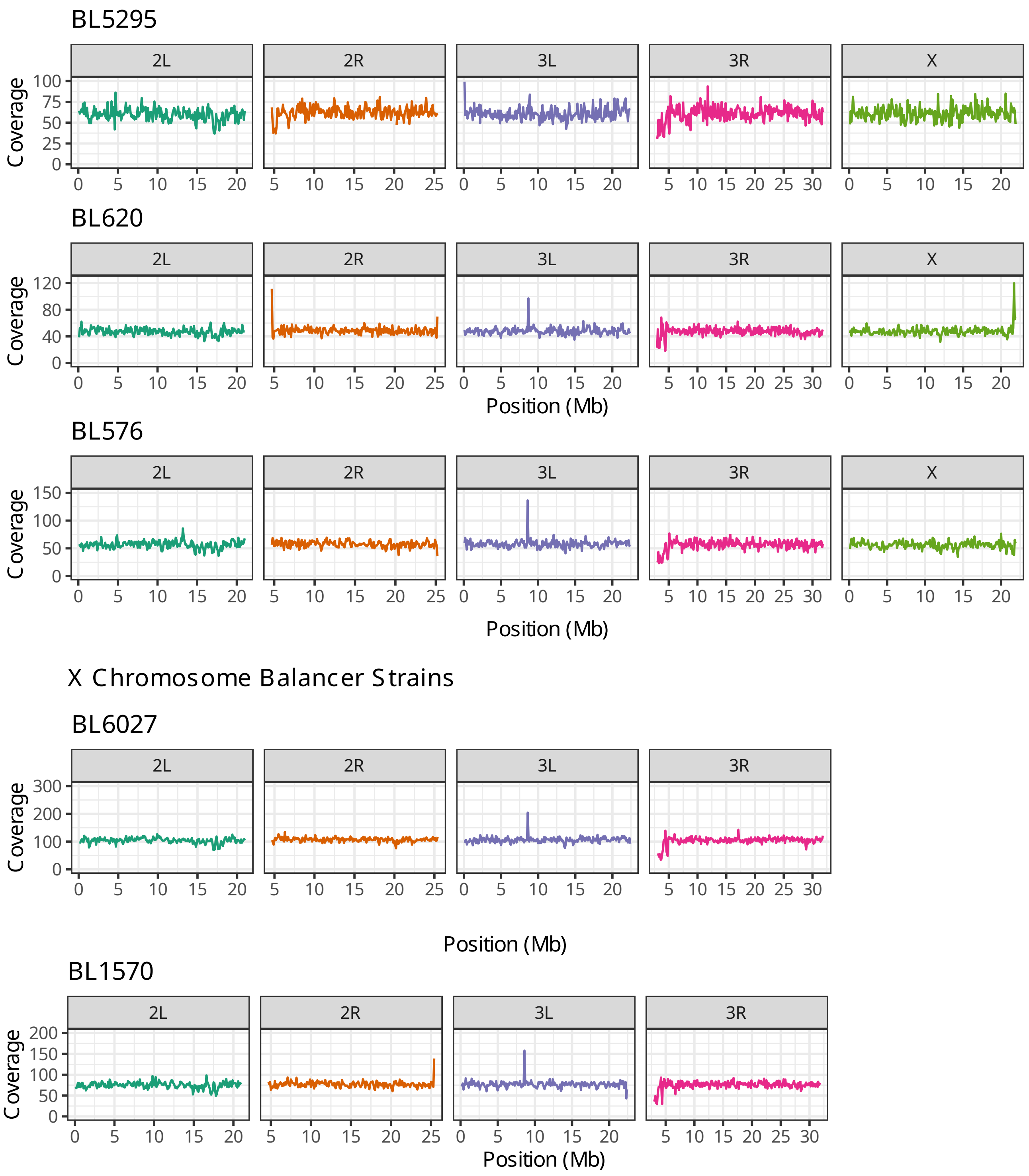

Supplemental Figure S1. Read-depth profiles after aligning raw reads to each strain's scaffolded assembly show near-uniform coverage across the major chromosome arms, with no extended dips or spikes indicative of collapsed repeats, consistent with the absence of major misassemblies.

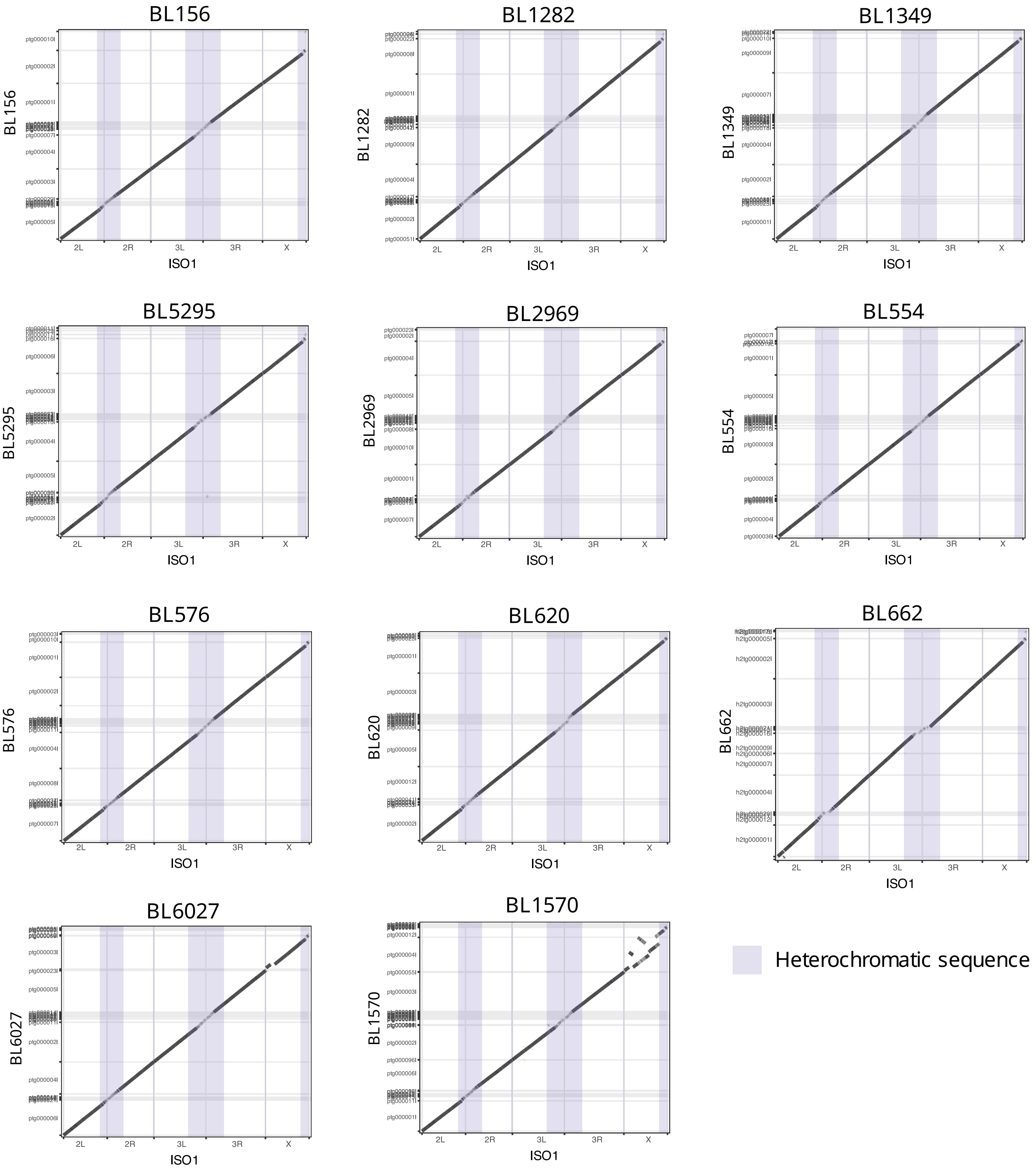

Supplemental Figure S2. Alignment dot plots between the ISO1 reference genome and the 11 *de novo* genome assemblies (unscaffolded contigs). Repeats are masked in both reference and query assemblies to show the overall alignment pattern.

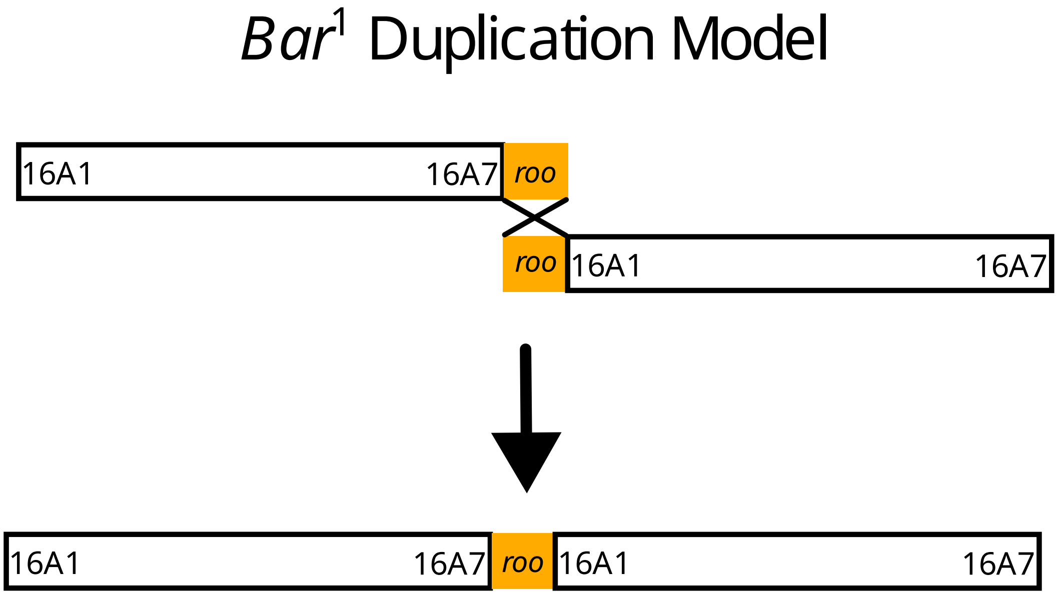

Supplemental Figure S3. Schematic illustrating the mechanism proposed by Sturtevant 1925 and Tsubota et al. 1989 for the origin of the Bar[1] duplication at cytological bands 16A1–16A7. A *roo* retrotransposon located near 16A7 is shown mediating non-allelic recombination between homologous regions, resulting in a tandem duplication of the 16A1–16A7 interval with the *roo* element positioned at the junction between the duplicated copies.

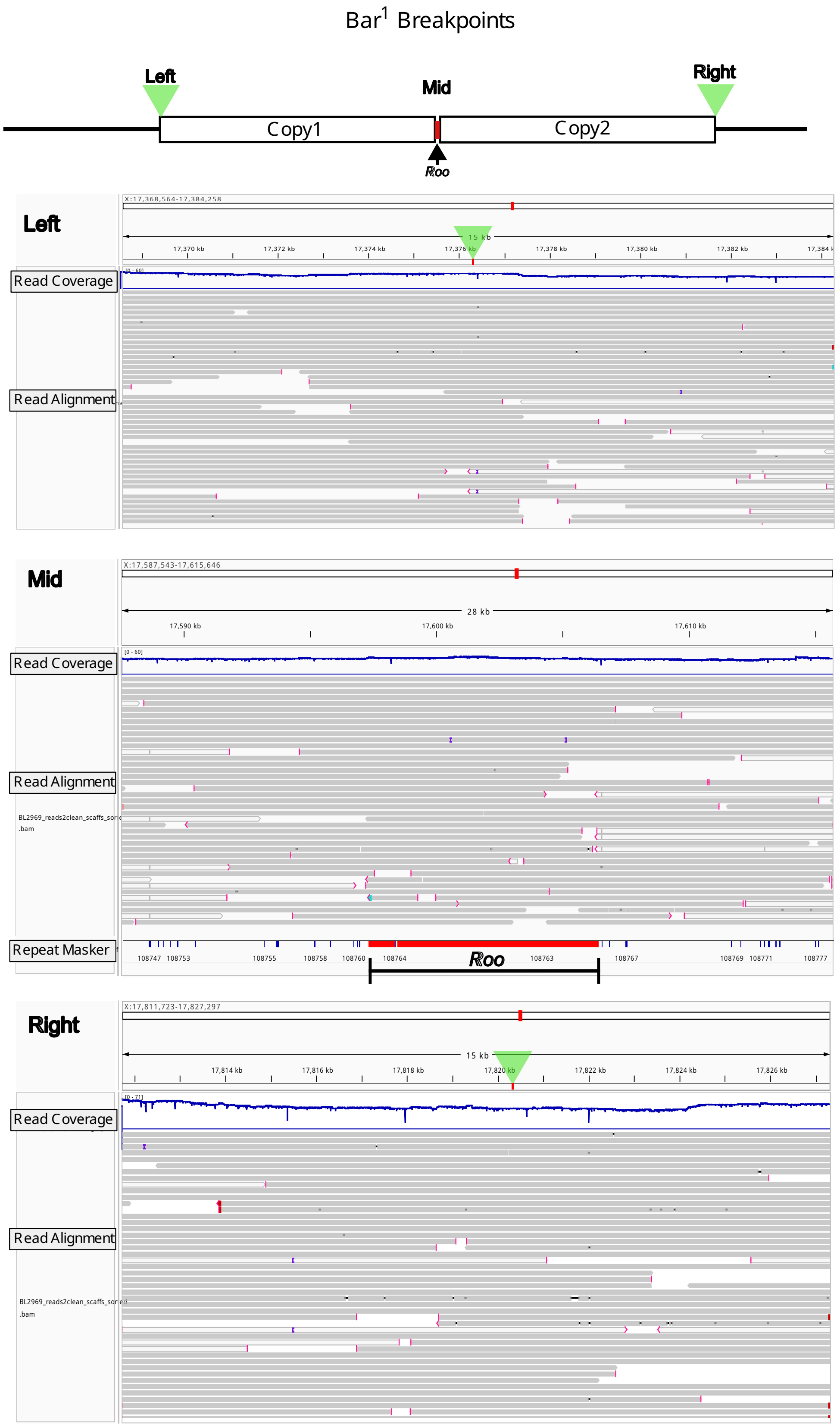

Supplemental Figure S4.. IGV alignment tracks showing long-read support for the breakpoints of the Bar[1] tandem duplication in the genome assembly of strain 2969. RepeatMasker annotation at the junction between the duplicated copies identifies a *roo* retrotransposon insertion, consistent with a model in which transposable elements facilitate unequal crossing over leading to duplication.

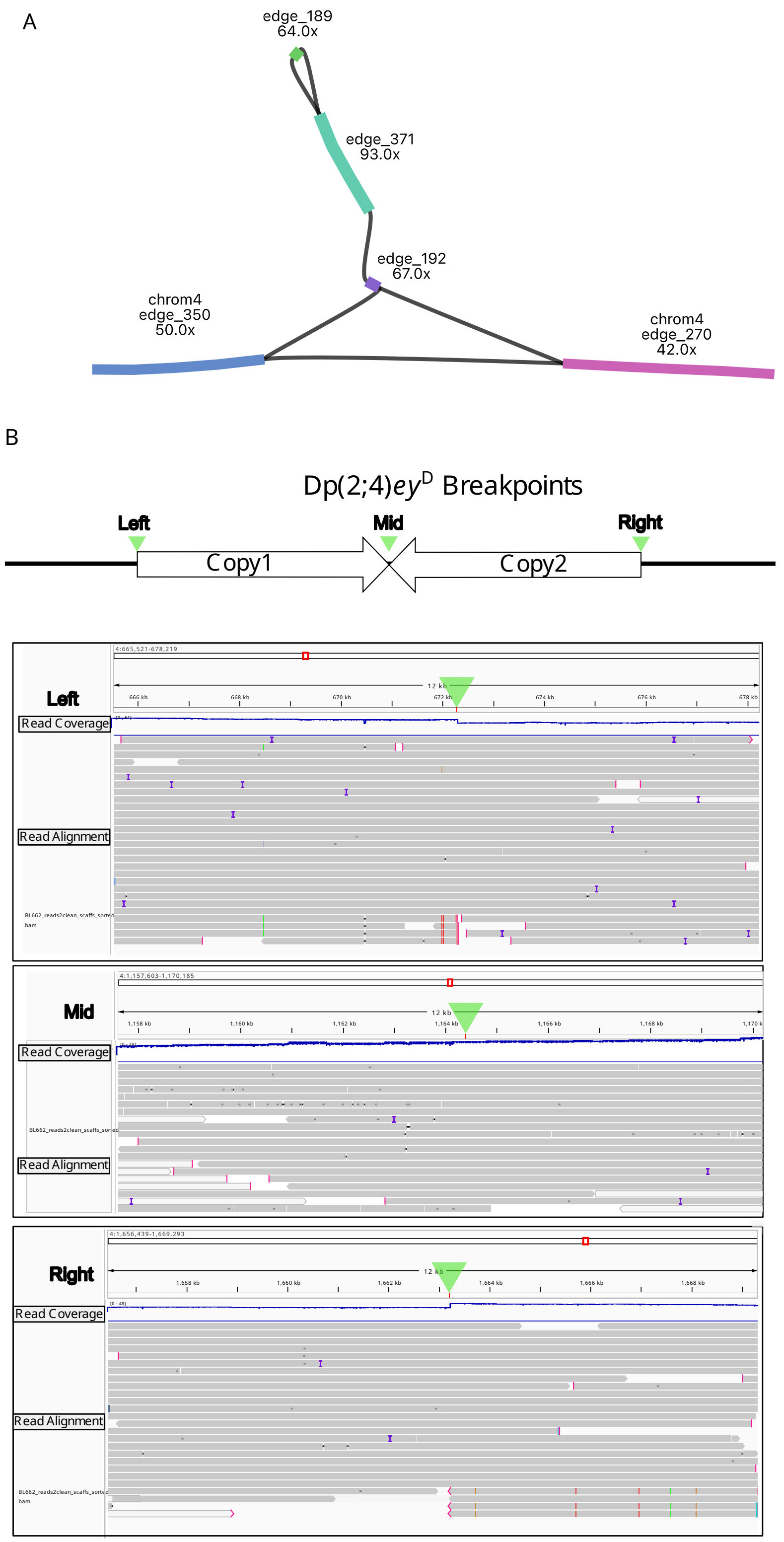

Supplemental Figure S5. A. Bandage plot showing the Flye repeat graph at the *ey*^{D} translocation-duplication region on chromosome 4 of strain 662. In the assembled genome, the duplication is collapsed, as indicated by the doubled coverage at edge_371. The duplication was manually resolved by exporting the path: edge_350 → edge_192 → edge_371 → edge_189 → edge_371 → edge_192 → edge_270.

B. Breakpoints were validated by confirming read support of the sequence in the corrected genome assembly.

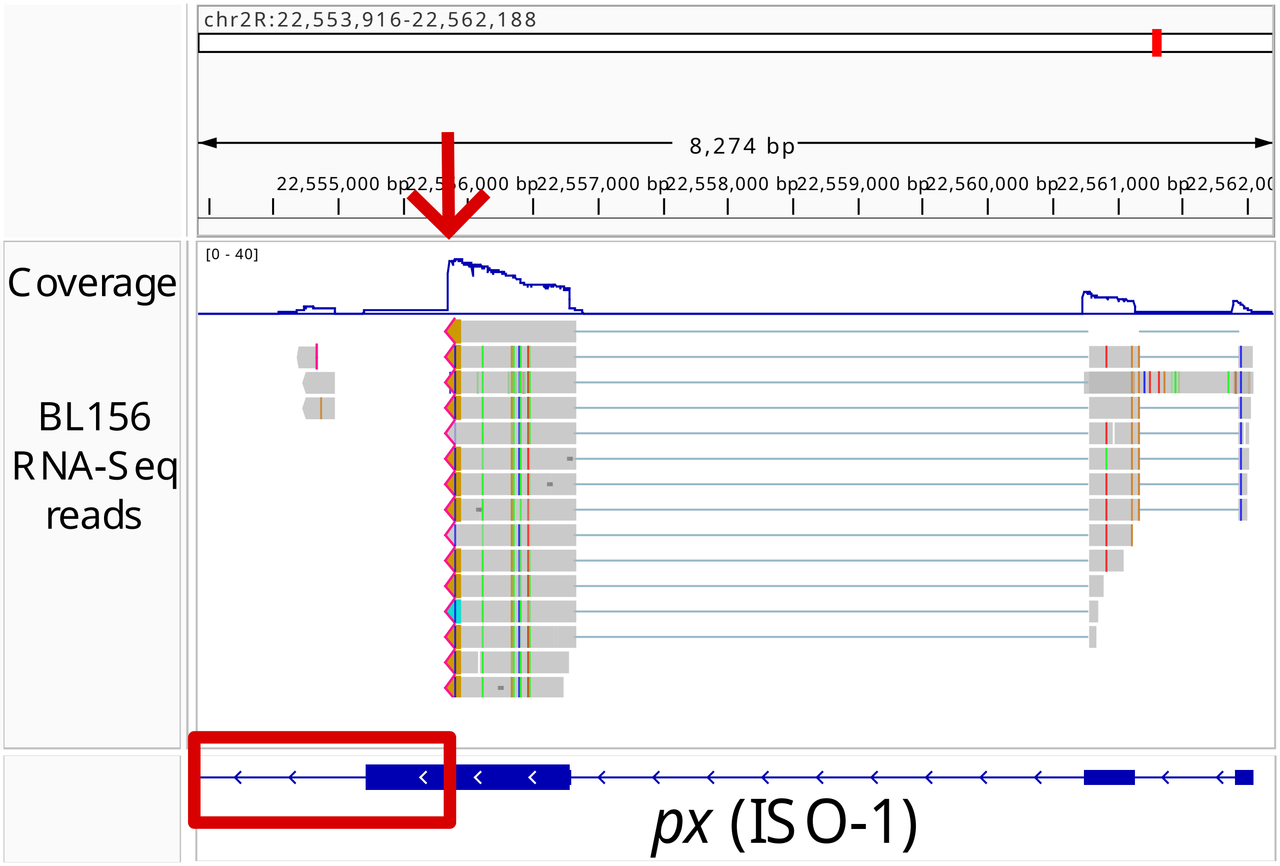

Supplemental Figure S6. Oxford Nanopore RNA-seq reads from strain 156 mapped to the ISO-1 reference across the *plexus* (*px*) locus on chromosome 2R. Read coverage (top) and individual RNA-seq alignments (middle) show a sharp termination of transcription at the site of the DM412 element insertion (red arrow). The boxed region highlights the truncated portion of the *px* transcript, with reads terminating in the middle of the exon annotated in the ISO-1 gene model (bottom).

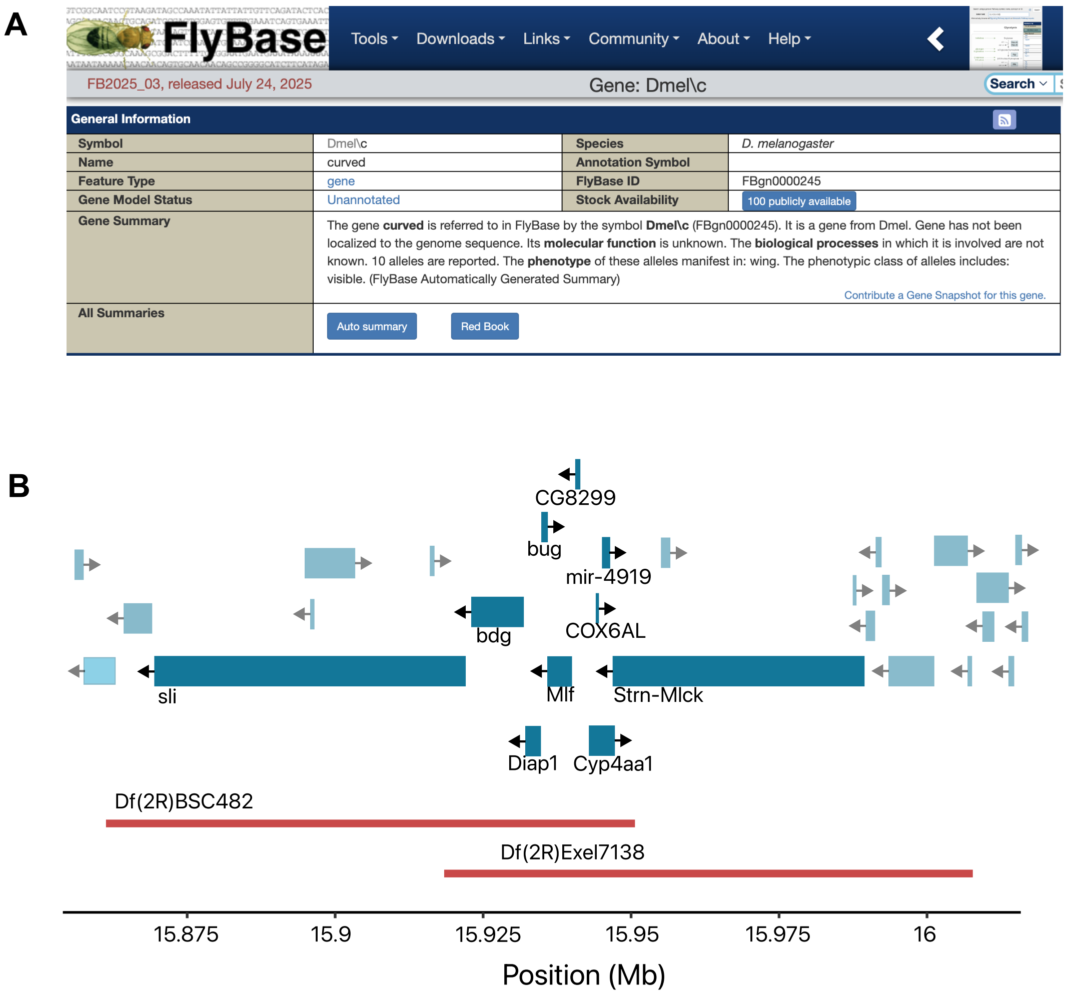

Supplemental Figure S7. A. FlyBase page for the gene curved, which is not yet mapped to a genomic location B. Deletion mapping from a previous study (Kahsai and Cook 2018) narrowed the location of *curved* to a set of 10 genes, with *Strn-Mlck* identified as the most probable candidate.

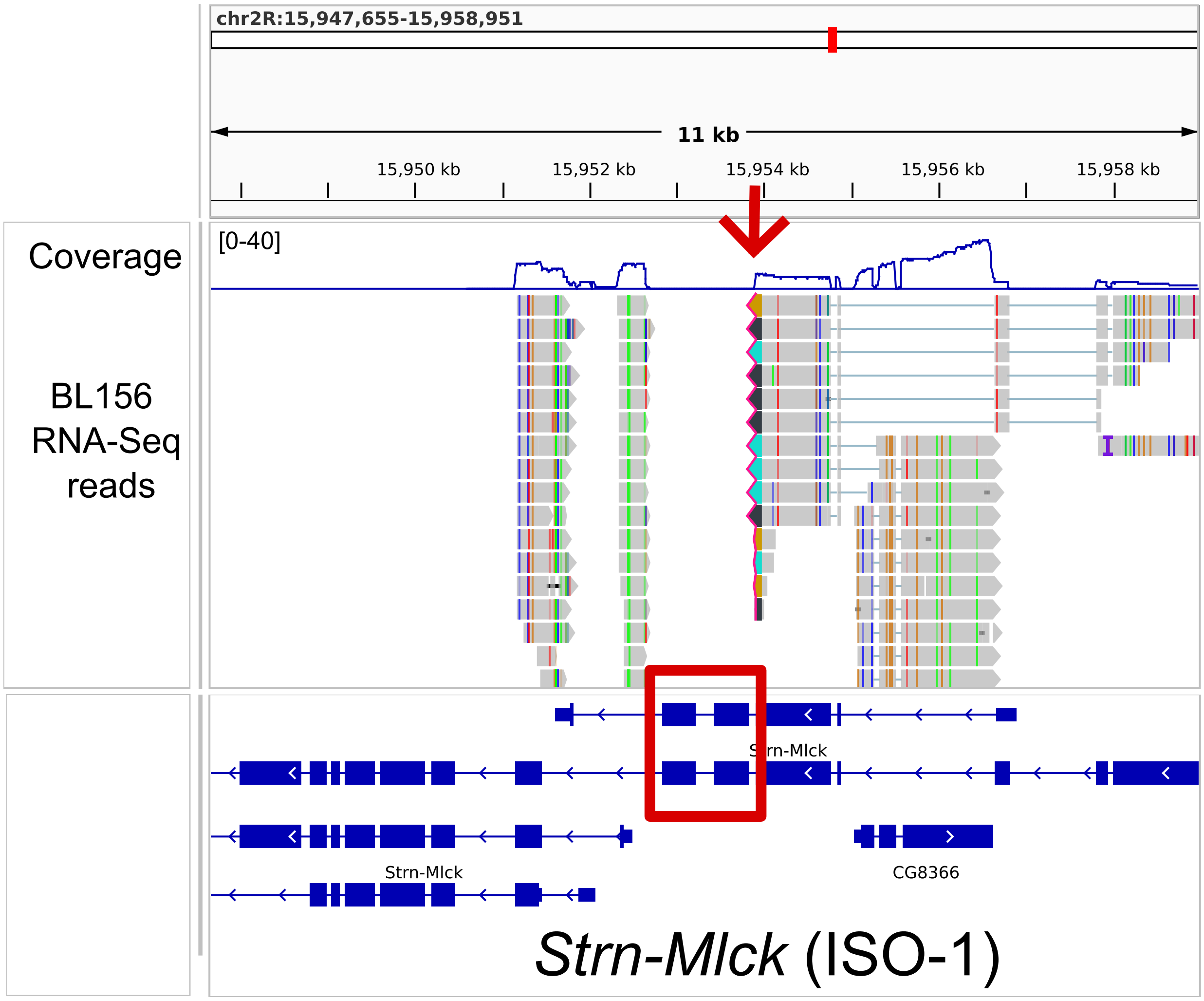

Supplemental Figure S8. Oxford Nanopore RNA-seq reads from strain 156 mapped to the ISO-1 reference across the *Strn-Mlck* locus on chromosome 2R. Read coverage (top) and individual RNA-seq alignments (middle) show termination of transcription at the site of the DM412 element insertion (red arrow). The boxed region highlights truncation of the *Strn-Mlck* transcript, with RNA-seq reads failing to extend into downstream exons annotated in the ISO-1 gene models (bottom).

A

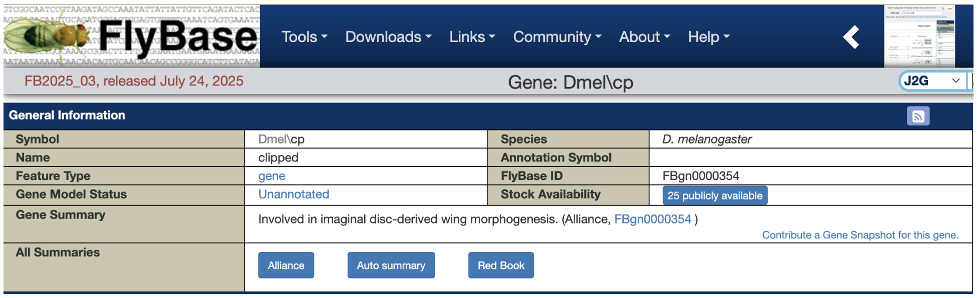

B

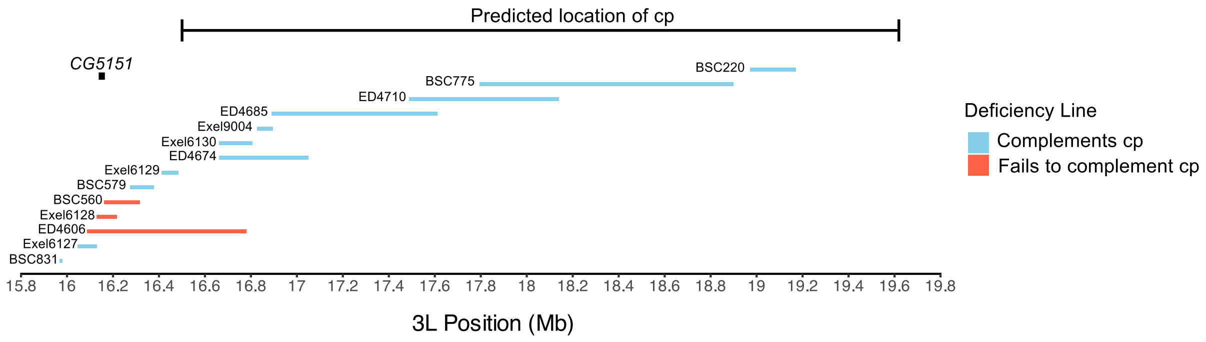

C

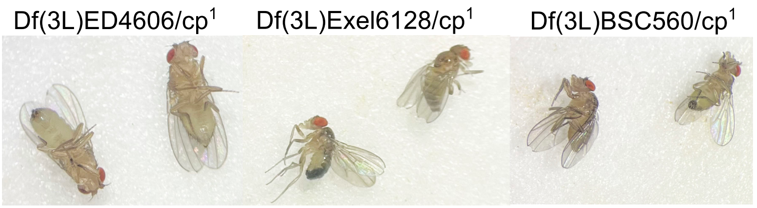

Supplemental Figure S9 . A. FlyBase page for the gene *clipped*, which is not yet mapped to a genomic location. B. Deletion mapping performed in this study using deficiency lines overlapping or located near the 3.12 Mb region predicted to contain *clipped*. Three deletions failed to complement the *clipped* phenotype, and, given their minimal overlap, the candidate region was narrowed to a 55 kbp interval (3L:16,162,336-16,217,328, Release 6) containing *CG5151*, a gene with a known role in wing development. C. Representative F1 males and females from deficiency line crosses showing failure to complement the cp^1 (clipped) phenotype.

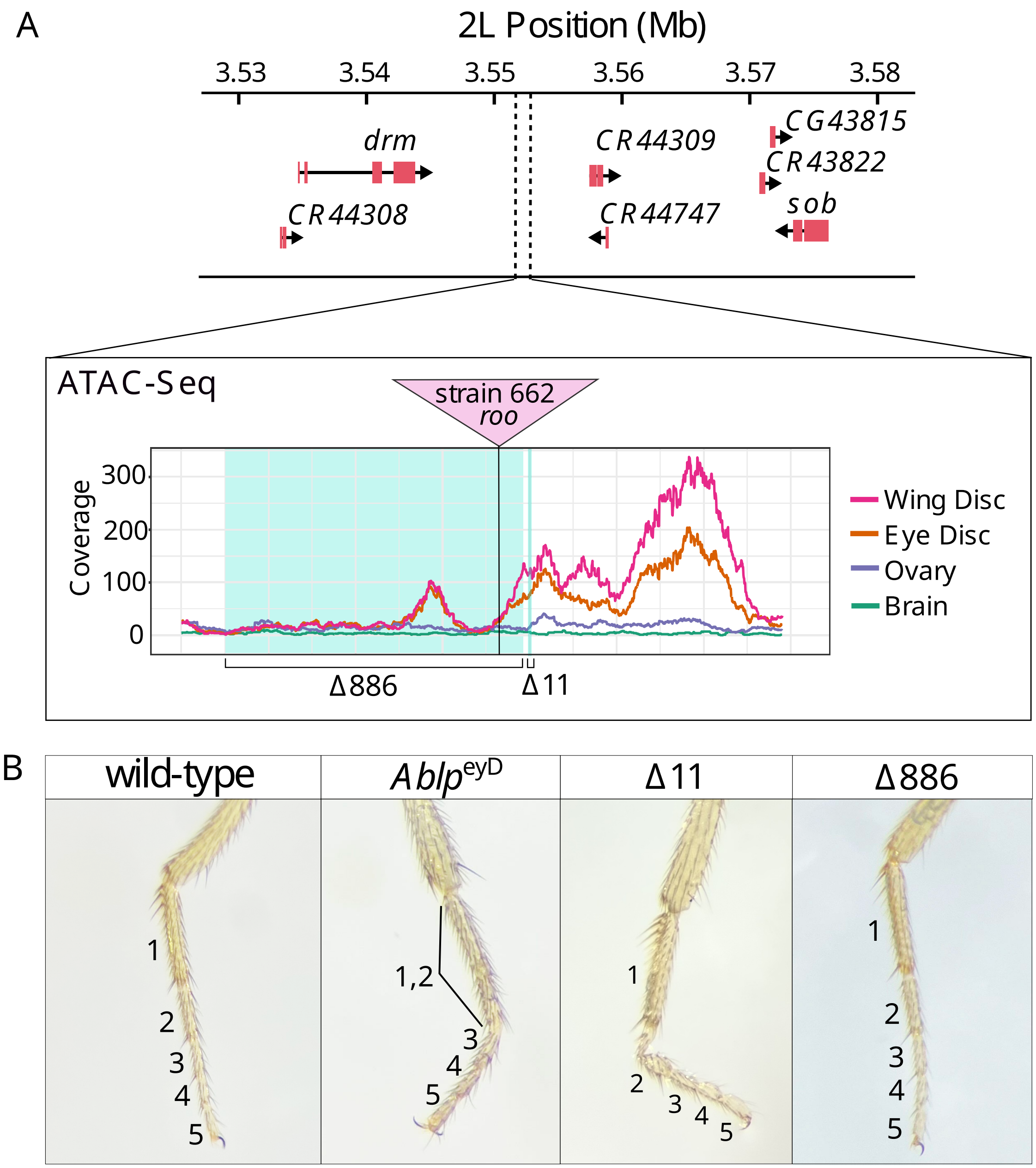

Supplemental Figure S10. A. Gene models and chromatin accessibility near the candidate *Ablp* locus on chromosome 2L. ATAC-seq data from the isogenic A6 strain reveal accessible chromatin in imaginal discs, but not adult tissues, at this locus. The highlighted regions indicate a complete deletion of a predicted regulatory sequence (Δ886) and a smaller off-target 11 bp deletion (Δ11) that falls within an ATAC-seq peak. A *roo* element insertion in this region is present in strain 662, which exhibits the *Ablp*^{eyD} phenotype. B. Tarsal segment phenotypes of wild-type, *Ablp*^{eyD}, homozygous Δ11, and homozygous Δ886 flies.

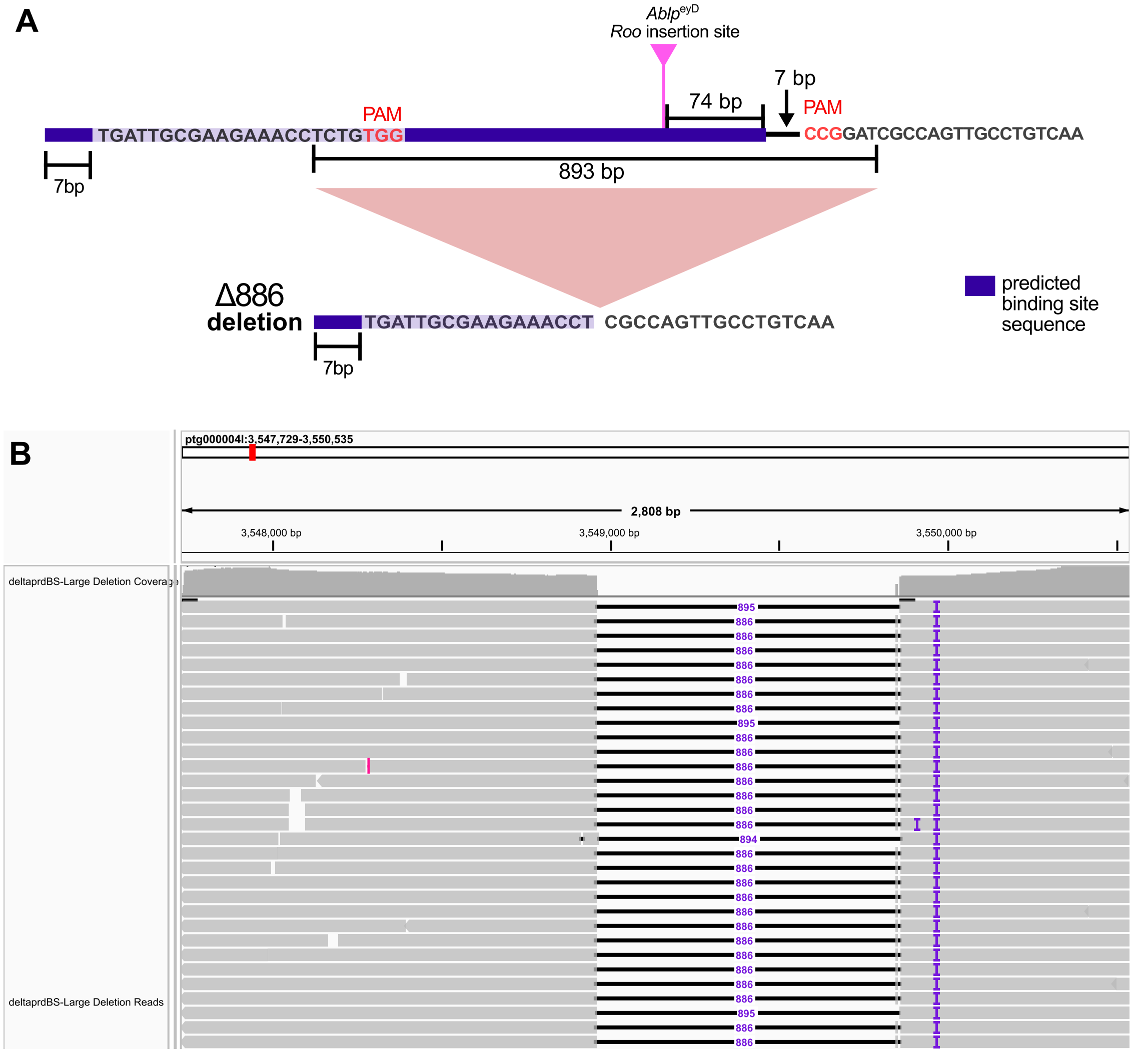

Supplemental Figure S11. A. Two guide RNA sequences were designed to produce full site deletion of the predicted binding site for *paired* (purple), in which an 8 kb *roo* element (pink) inserts in strain 662, which carries the *Ablp*^{eyD} phenotype. B. Reads from PCR amplification of the targeted locus were aligned to the 54591 genome, showing an 886 bp deletion between the two guide RNA sites.

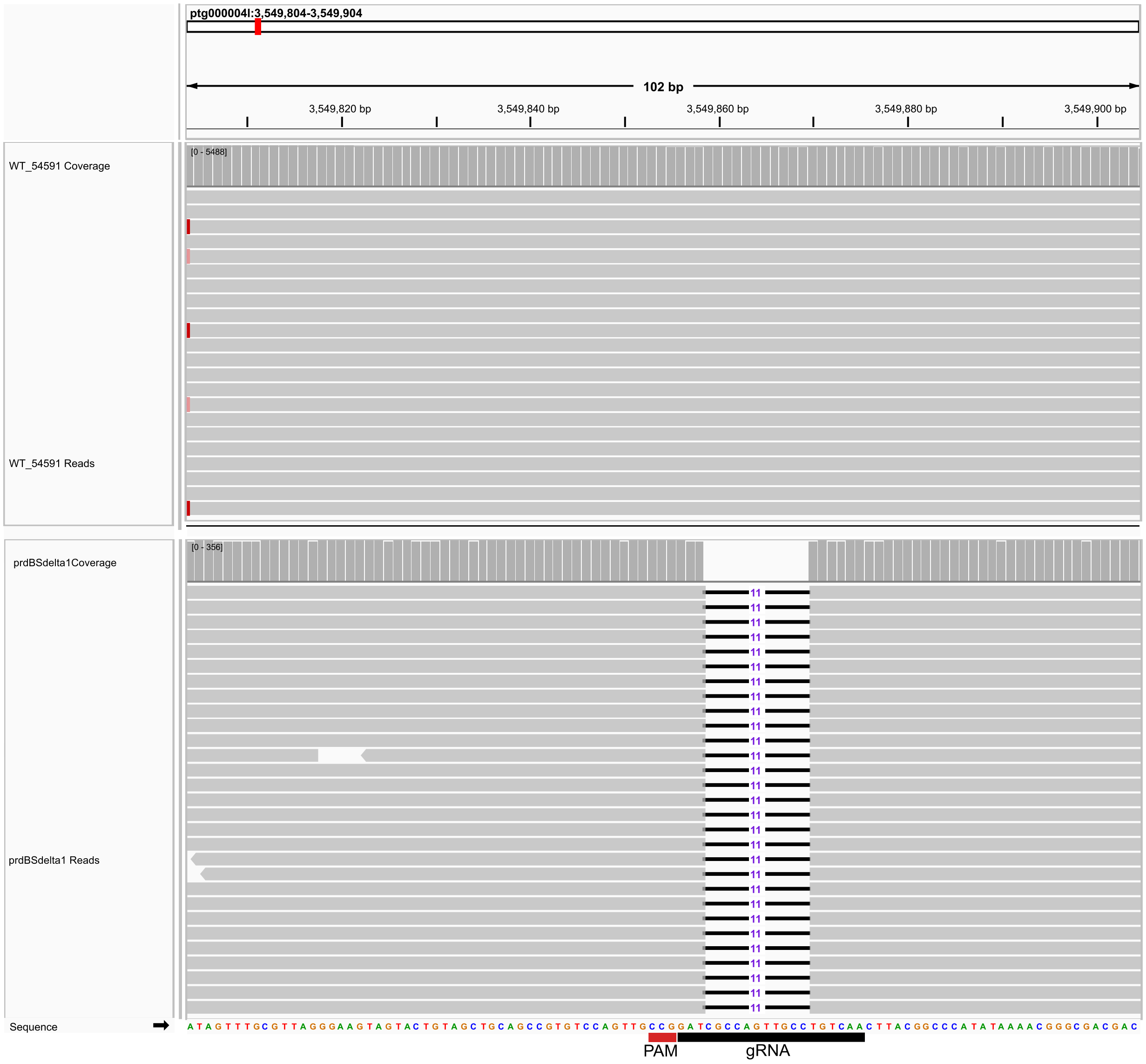

Supplemental Figure S12. Reads from PCR amplification of the targeted locus were aligned to the 54591 genome. Top: From a wild-type individual, reads show alignment with no indels across the guide RNA (gRNA) target and PAM, consistent with the 54591 sequence. Bottom: Edited individual displaying the leg-joint phenotype: reads show an 11-bp deletion spanning the predicted Cas9 cut site (3 bp upstream of the PAM), here designated Δ11.

A B C

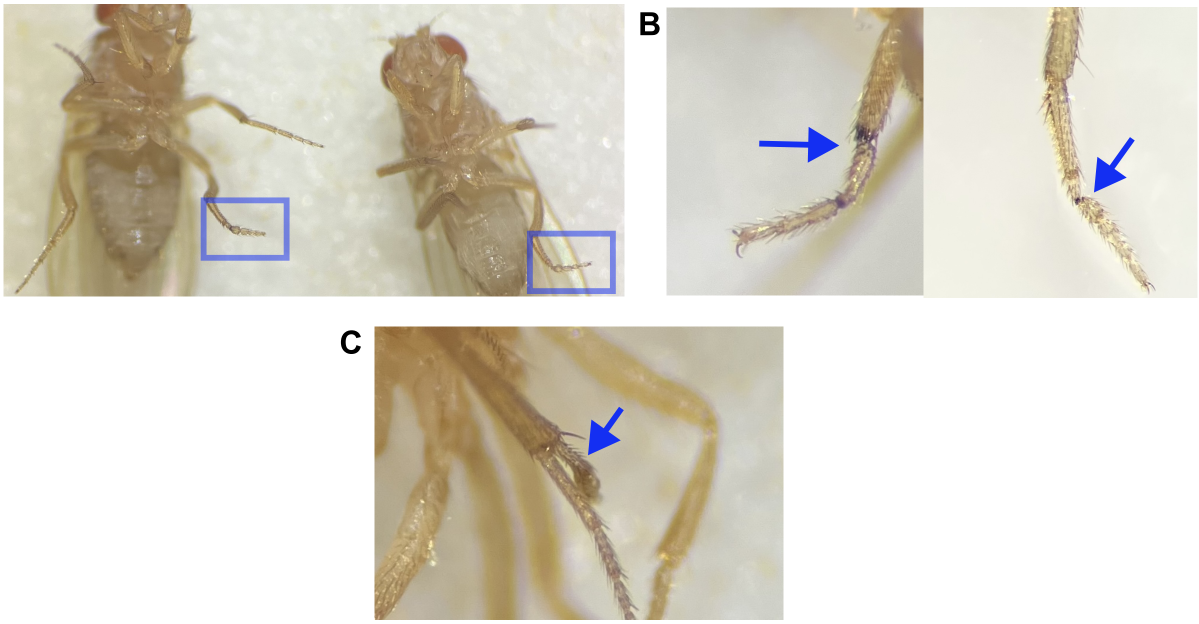

Supplemental Figure S13. Leg phenotypes of transgenic flies carrying CRISPR-mediated deletion, Δ11. A. Two females displaying the most common phenotype, a pinched tarsal segment. B. Pinched tarsal segments often turned black, indicative of necrotic tissue. C. A single female exhibited a duplicated tarsal segment growing from the tibia.

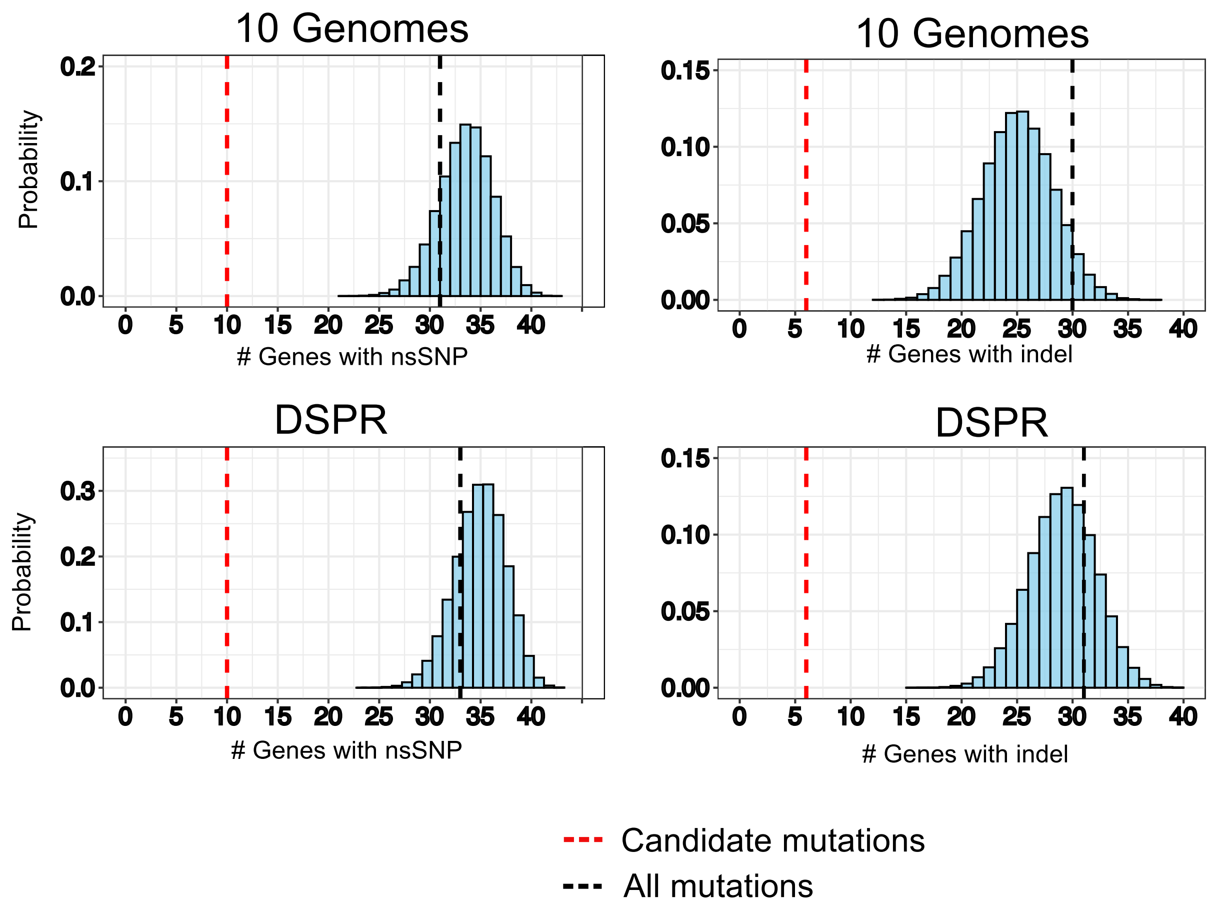

Supplemental Figure S14. Monte Carlo simulations testing enrichment of nonsynonymous SNPs (nsSNPs; left) and indels affecting 2-10 nucleotides (right) among the 43 genes associated with the 50 visible phenotypes analyzed in this study. Distributions show the expected number of genes harboring at least one mutation when randomly sampling 43 genes matched for gene length from either the 10-genome variant map (top) or the DSPR variant map (bottom). Dashed black lines indicate the observed number of genes containing any nsSNP or indel, while dashed red lines indicate the number of genes containing candidate causative mutations. The number of marker genes affected by nsSNPs does not differ significantly from the null expectation (10 genomes: p-value = 0.168; DSPR: p-value = 0.243), and similarly, small indels show no significant enrichment (10 genomes: p-value = 0.109; DSPR: p-value = 0.387). In all cases, the number of marker genes harboring candidate causative nsSNPs or indels is significantly lower than expected under the null, indicating that these mutation types are underrepresented among our candidate mutations.

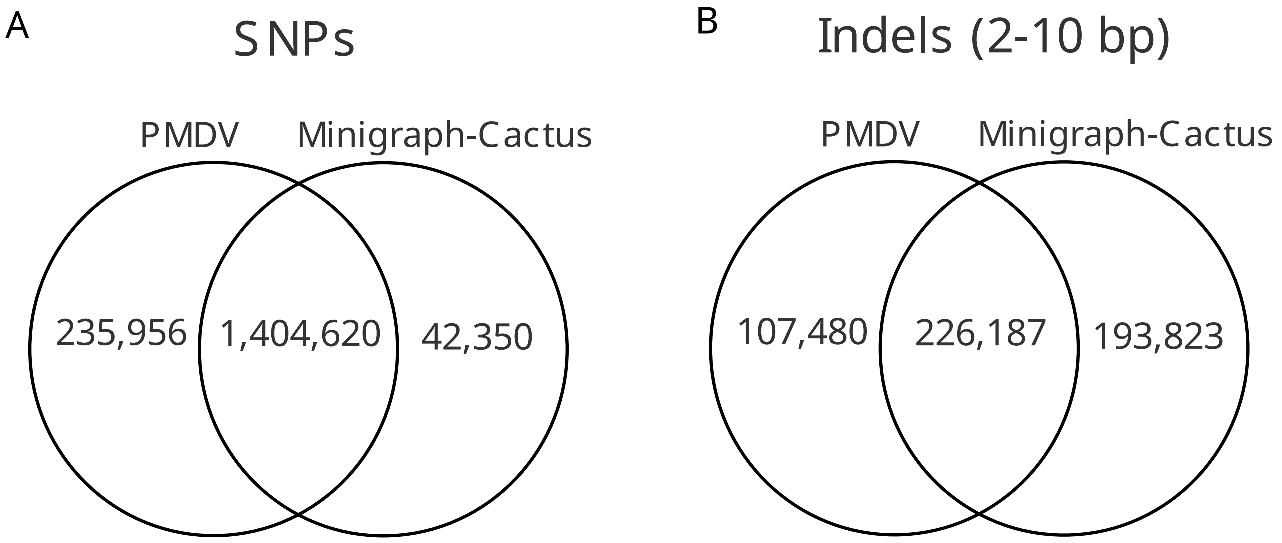

Supplemental Figure S15. Venn diagrams comparing (A) SNP and (B) small indel (2-10bp) calls generated by the PEPPER-Margin-DeepVariant (PMDV) pipeline and the Minigraph-Cactus pangenome graph. To ensure a fair comparison, all variants were normalized and left-aligned using bcftools norm prior to intersection with bcftools isec.

Supplementary Text

Additional Information for Previously Uncharacterized Candidate Mutations

aristaless, al[1]

Strain 156 has a 10 bp deletion in the last coding exon of *aristaless*, the only disruptive mutation unique to this strain.

asteroid, ast[1]

The breakpoints of this mutation were approximated in a previous publication (Higson et al. 1993) with restriction mapping and cloning. Higson predicted that the mutation was an insertion greater than 21 kb, about 3 kb upstream of the transcription start site of the gene. The genome assembly shows a complete duplication of the *asteroid* gene and a 33.8 kb insertion consisting of *HMS-Beagle*, *gypsy*, and *roo* elements between the copied region.

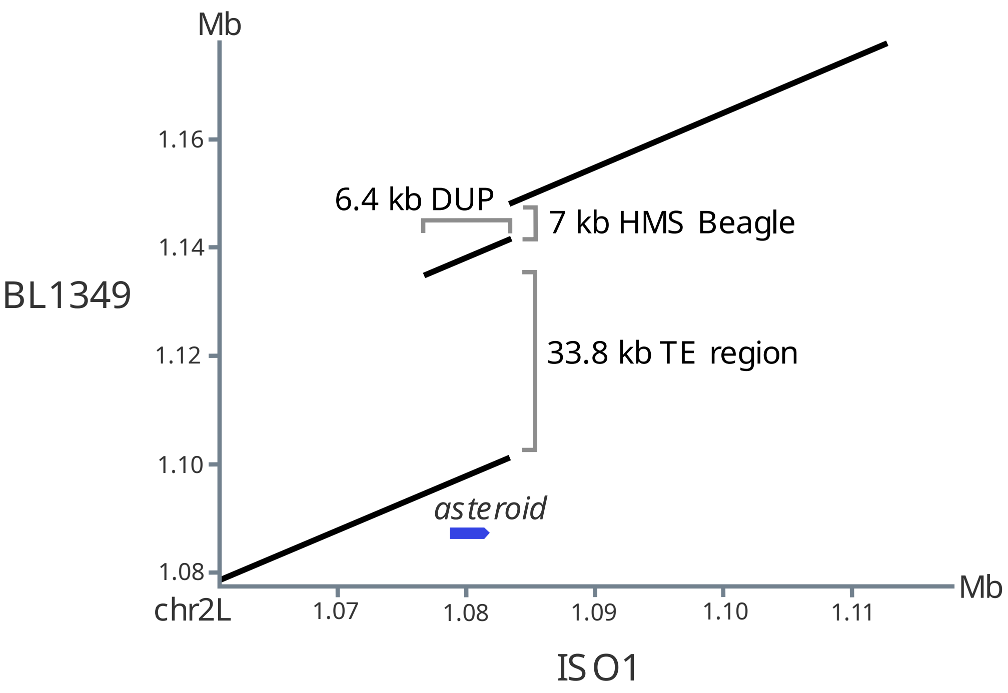

Diap1[th-1] also known as *thread*[1]

Both 620 and 576 show the same *thread* phenotype, where the aristae are lacking lateral branches. Despite this, the strains lack any shared mutations in or near the gene *Diap1* that are not present in other strains lacking the phenotype. Strain 576 has a nonsynonymous SNP, however, this is not present in 620. Previous study of *thread* mutants postulated that this phenotype might be a regulatory mutation, as it seems to affect only one of the transcript variants, possibly one with a role in aristal antennae development (Cullen and McCall 2004). We do not observe any SVs affecting the *Diap1* gene, so we infer that a SNP or small indel could be the candidate mutation. 620 and 576 both have several intronic SNPs and small indels in close proximity to each other, which affect a predicted regulatory sequence (https://flybase.org/reports/FBsf0000921025).

ebony, e[s] or *sooty*
Strains 576 and 554, which both carry the *sooty* allele, have a nonsynonymous SNP in the *ebony* gene which is not present in strains lacking the phenotype.
echinoid, ed[1]
Strain 1349 has a 7.8 kb insertion of *gypsy* and *Stalker* transposable elements, which is not present in other strains lacking the phenotype.
garnet, g[1] and g[2]
Southern Blot analysis of g[1] and g[2] mutants (Lloyd et al. 1999), indicated that the g[1] allele is due to a large insertion and g[2] is likely a small point mutation. We find that strain 6027, which carries g[1], has a 7.4 kb Blood element insertion into an intron. Strains 5295 and 1570 are both g[2] and share two coding variations: a 3bp in-frame deletion and nonsynonymous SNP.
in[1]
Strain 620 has a nonsynonymous SNP at 3L: 20,369,329 which is the only disruptive mutation to the *in* gene that is unique to this strain
miniature m[74f]
Strain 1282 has several SNPs and indels unique to the strain in the miniature gene, which consist of 3 bp indels in the intron, possibly affecting a regulatory element (https://flybase.org/reports/FBsf0000873514).
plexus, px[1]
Strain 156 harbors several large mutations in the *plexus* gene, including a 3.5 kb "deletion" corresponding to the absence of a TE present in the reference genome, a 7.3 kb *Quasimodo* insertion within an intron, a 1.5 kb partial duplication of a coding exon, and a 7.4 kb *DM412* insertion located between the duplicated segments. While the *Quasimodo*, duplication, and *DM412* insertions are unique to strain 156, we predict that the variants disrupting the coding sequence are the likely causative mutations.

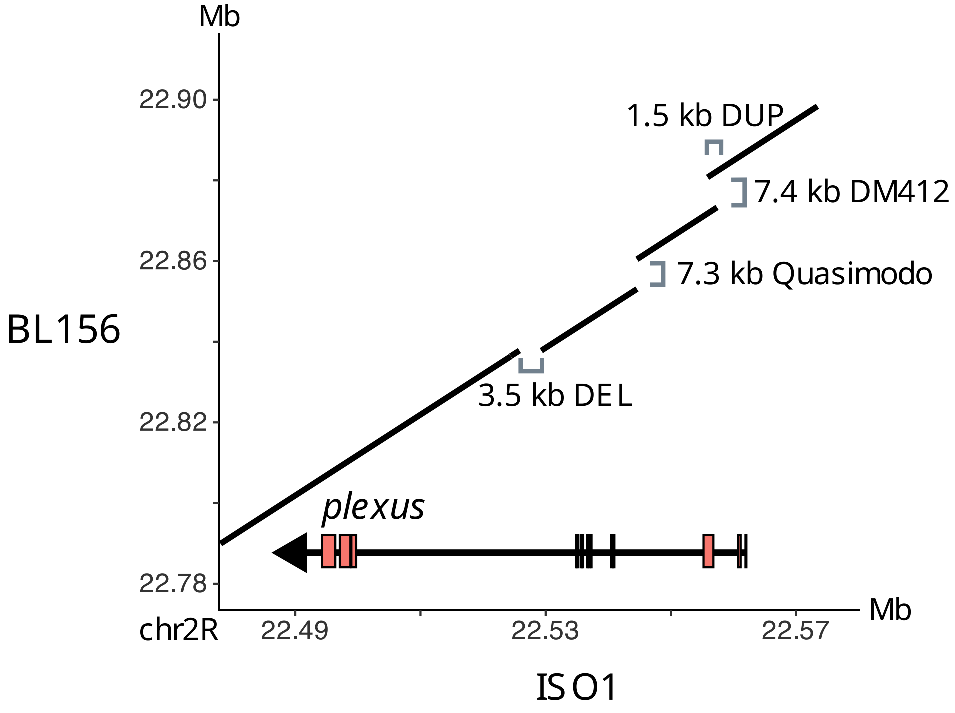

ruby, rb[1]
Strain 6027 has a 7.8kb gypsy1/Stalker2 insertion into a coding exon of *ruby* which is found only in this strain.
sd[1]
Strain 1282 has a 22 bp deletion which removes part of the coding sequence from a single transcript (FBtr0346799), and is unique to this strain.
stripe, sr[1]
Strains 576 and 554 share four SNPs in the 5' UTR, two SNPs in an intron, and a 4 bp deletion in an intron, which are not found in other strains lacking the *stripe* phenotype. No SVs were identified within the gene or within 1 kb so we inferred that one or more of the SNPs or indels are the causative mutation.
Ab(1)os-s (upd1[os-s],upd3[os-s])
Strain 1282 has three unique, likely disruptive mutations to the gene *upd*^{3}, including a disruptive in-frame (3bp) deletion, and a nonsynonymous SNP. The gene *upd1* has no obviously disruptive mutations.

Supplemental Tables

Supplemental Table S1: Bloomington stock numbers of the sequenced strains and their genotype

Strain Name	Genotypes
1282	Ab(1)os[s], cm[1] m[74f] sd[1] upd1[os-s] upd3[os-s]
1349	ast[1] dpp[d-ho] ed[1] dpy[ov1] cl[1]
156	al[1] dpy[ov1] Adc[b-1] pr[1] c[1] px[1] speck[1]
1570	Df(1)os[o], y[1] pn[1] w[1] cm[1] ct[6] sn[3] oc[1] ras[2] v[1] dy[1] g[2] f[1] upd1[os-o] upd3[os-o]/FM6
2969	Bar[1]
5295	y[1] w[1118] sn[3] v[1] g[2] f[1]/Dp(1;Y)y[+]
554	p[p] Ubx[bx-1] sr[1] e[s]
576	ru[1] hry[1] Diap1[th-1] st[1] cu[1] sr[1] e[s] ca[1]
6027	y[1] ac[1] sc[1] pn[1] w[1] rb[1] cm[1] ct[1] sn[3] ras[4] v[1] m[1] g[1] f[1] car[1]/FM6
620	Diap1[th-1] st[1] cp[1] in[1] kni[ri-1] p[p]
662	sv[de]/Dp(2;4)ey[D], Ablp[eyD]: ey[D]

Supplemental Table S2. Sequencing statistics

Strain Name	SRE kit used?	DNA sequenced (ug)	Bases sequenced (Gbp)	Read N50 (bp)	%duplex	average coverage
1282		287.92	8.86	11,520	7.04	63.30
1349	yes	245.88	15.77	22,059	9.12	112.62
156		296.16	19.82	8,566	7.11	141.60
1570		254.10	12.65	12,614	6.11	90.39
2969	yes	262.14	10.29	32,293	8.90	73.51
5295	yes	286.30	9.89	24,636	10.05	70.66
554		203.52	11.51	17,067	7.90	82.19
576	yes	275.20	9.45	20,883	7.37	67.50
6027		265.44	16.39	9,272	6.25	117.08
620		285.48	7.74	17,712	11.85	55.26
662		265.80	15.63	9,597	6.33	111.68

Supplemental Table S3. Detailed assembly statistics

Strain Name	Contigs			Scaffolds			
	Length (bp)	#	N50 (bp)	Length (bp)	#	N50 (bp)	Ns per 100kbp
1282	146,015,810	56	24,071,413	146,017,810	36	26,597,705	1.37
1349	151,221,306	49	24,177,558	151,223,406	28	29,439,780	1.39
156	154,310,040	36	23,763,809	154,311,840	18	29,305,395	1.17
1570	159,240,532	96	21,808,979	159,243,332	68	28,770,337	1.76
2969	150,107,498	34	23,786,490	150,109,198	17	29,658,738	1.13
5295	154,080,537	53	24,308,267	154,082,637	32	29,850,755	1.36
554	150,375,436	51	24,063,459	150,377,736	28	28,797,642	1.53
576	151,814,353	44	21,703,497	151,816,453	23	29,131,788	1.38
6027	151,358,994	69	23,909,075	151,360,994	49	27,884,710	1.32
620	151,934,778	51	24,293,528	151,936,978	29	27,622,789	1.45
662	153,488,684	45	18,537,079	153,491,084	21	28,981,392	1.56

Supplemental Table S4. *clipped*[1], *Ablp*[eyD], and *curved*[1] strains for barcoded sequencing

Strain Name	Genotype	Allele of Interest
1408	Diap1[th-1] st[1] cp[1] in[1] kni[ri-1] Kg[V] Ki[1] p[p]/TM3, ry[RK] Sb[1] Ser[1]	cp[1]
3308	Diap1[th-1] st[1] cp[1] in[1] kni[ri-1] bcd[2] hb[9]/TM3, Sb[1] Ser[1]	cp[1]
3313	Diap1[th-1] st[1] cp[1] in[1] kni[ri-1] p[p] sim[8]/TM3, Sb[1]	cp[1]
3407	ru[1] hry[1] st[1] cp[1] in[1] kni[ri-1] Antp[Scx] p[p] cu[1] e[1]/TM3, Sb[1]	cp[1]
3309	Diap1[th-1] st[1] cp[1] in[1] kni[6] kni[ri-1] p[p] e[1] tll[1]/TM3, Sb[1] Ser[1]	cp[1]
3363	Df(3R)9A99, Diap1[th-1] st[1] cp[1] in[1] kni[ri-1] kni[6] cu[1] e[s] ca[1]/TM3, ry[RK] Sb[1] Ser[1]	cp[1]
4343	ci[Ce-2]/Dp(2;4)ey[D], Ablp[eyD]: ey[D]	Ablp[eyD]
4760	pan[3]/Dp(2;4)ey[D], Ablp[eyD]: ey[D]	Ablp[eyD]
55237	y[1]; PBac{y[+mDint2]=HpaI-GFP.A}ci[YD1062]/Dp(2;4)ey[D], Ablp[eyD]: ey[D]	Ablp[eyD]
605859	y[*] w[*]; P{?SUPor-P}Asator[exc]/Dp(2;4)ey[D], Ablp[eyD]: ey[D]	Ablp[eyD]
6308	y[1] w[*]; P{w[+mC]=lacW}Dplac ci[Dplac]/Dp(2;4)ey[D], Ablp[eyD]: ey[D]	Ablp[eyD]
850	T(1;4)w[m5]/Dp(2;4)ey[D], Ablp[eyD]: ey[D]	Ablp[eyD]
1087	l(4)102CDe[1]/Dp(2;4)ey[D], Ablp[eyD]: ey[D]	Ablp[eyD]
93031	w[1118]; sv[7]/Dp(2;4)ey[D], Ablp[eyD]: ey[D]	Ablp[eyD]
250	c[1]	c[1]
4195	Adc[b-1] pr[1] c[1] px[1] speck[1]	c[1]
5554	al[1] Adc[b-1] l(2)49Dc[3] c[1]/CyO	c[1]
1882	w[*]; al[1] Adc[b-1] c[1] speck[1]	c[1]
8210	P{ry[+t7.2]=neoFRT}42D cn[1] c[1] px[1] speck[1]	c[1]
5753	w[*]; P{w[+mW.hs]=FRT(w[hs])}G13 c[1] px[1] speck[1]	c[1]

Supplemental Table S5. *Clipped* deletion mapping strains

Strain Name	Deficiency Line	Start (3L, ISO1r6)	End (3L, ISO1r6)	Complemented cp[1]?
29021	Df(3L)BSC831/TM6C	15,967,776	15,979,964	Yes
7606	Df(3L)Exel6127	16,046,940	16,129,654	Yes
8078	Df(3L)ED4606	16,087,484	16,780,123	No
7607	Df(3L)Exel6128	16,129,654	16,217,328	No
25122	Df(3L)BSC560/TM6B	16,162,336	16,318,079	No
25413	Df(3L)BSC579/TM6C	16,274,238	16,378,604	Yes

7608	Df(3L)Exel6129	16,411,693	16,483,557	Yes
8098	Df(3L)ED4674	16,661,284	17,049,418	Yes
7609	Df(3L)Exel6130	16,661,291	16,806,648	Yes
7937	Df(3L)Exel9004/TM6B	16,826,256	16,895,610	Yes
64121	Df(3L)ED4685	16,891,076	17,612,170	Yes
8100	Df(3L)ED4710	17,487,463	18,139,299	Yes
27347	Df(3L)BSC775	17,795,144	18,898,326	Yes
9697	Df(3L)BSC220	18,972,562	19,171,268	Yes

Supplemental Table S6. TSV with coordinates and description of candidates mutations for cp^1
Supplemental Table S7. TSV with coordinates and description of candidate mutations for all 50 phenotypes.

Supplemental Table S8. References for deleterious effects of visible phenotypes

#	**Mutation**	**Origin**	**Phenotypic Defects**	**Defects References**
1	ac[1]	spontaneous	lack of sensory bristles	DOI:10.1007/BF01681532
2	Adc[b-1]	spontaneous	dark color, other cuticular defects and target recognition	DOI:10.1016/j.gene.2005.03.013
3	al[1]	spontaneous	claws and arista are reduced in size, females exhibit reduced mating success	DOI: 10.1242/dev.127.20.4315, 10.1016/s0003-3472(71)80025-8, DOI:10.1016/s0003-3472(71)80025-8
4	ast[1]	spontaneous	abnormal eye morphology, muscular defects	DOI: 10.1242/dev.120.7.1731, DOI: 10.1242/dev.00843
5	c[1]	spontaneous	wings curved, flightless	Lindsley, D.L., Grell, E.H. (1968)
6	ca[1]	spontaneous	reduced mating success in males	DOI: 10.1038/hdy.2011.60
7	car[1]	X Ray	reduced lifespan in males	DOI:10.1534/genetics.106.065011
8	cl[1]	spontaneous	no other defects observed besides abnormal eye color	
9	cm[1]	spontaneous	males have reduced lifespan, retinal response to light defective	DOI:10.1534/genetics.106.065011, DOI:10.1371/journal.pbio.1001847
10	cp[1]	spontaneous	clipped wing margins, reduced viability	Lindsley, D.L., Grell, E.H. (1968)
11	ct[1]	spontaneous	defective wing margins and deformed antennae	DOI: 10.1242/dev.124.17.3241
12	ct[6]	spontaneous	defective wing margins and abnormal gravitaxis	DOI:10.1093/genetics/160.4.1481, DOI:10.1007/BF01074308
13	cu[1]	spontaneous	wings are curved upward, defective	DOI:10.1266/jjg.31.321

14	Diap1[th-1]	spontaneous	aristae missing lateral branching, abnormal gravitaxis	DOI:10.1007/BF01074308,DOI:10.1007/BF01074308,DOI:10.1016/0092-8674(95)90150-7
15	dpp[d-ho]	spontaneous	held out wings, defects on the wing blade	DOI:10.1101/gad.4.11.2011,
16	dpy[ov1]	spontaneous	defective, dumpy wings	DOI:10.1016/j.devcel.2015.06.019
17	dy[1]	spontaneous	reduced wing size	FlyBase page FBal0003265
18	e[s]	spontaneous	abnormal body color, defects in activity and visual response	DOI:10.3109/01677069109066213
19	ed[1]	spontaneous	eyes rough and misrotated ommatidia	DOI:10.1534/g3.117.300289, DOI:10.1242/dev.038422
20	f[1]	spontaneous	abnormal bristles, reduced response to courtship sounds	DOI:10.1177/000348940811701106,
21	g[1]	spontaneous	reduced lifespan in males, disturbed walking and orientation	DOI:10.1534/genetics.106.065011,DOI:10.1093/genetics/155.1.213
22	g[2]	spontaneous	abnormal courtship behavior, possibly affects vision	DOI:10.1093/hmg/ddp555
23	hry[1]	spontaneous	ectopic wing bristles, minor defects in segmentation	DOI:10.1093/genetics/111.3.463, DOI: 10.1002/j.1460-2075.1993.tb05854.x
24	in[1]	spontaneous	abnormal hair polarity, incomplete extra leg joints	DOI:10.1093/genetics/160.4.1535
25	kni[ri-1]	spontaneous	missing wing vein, wings are warped and blunt	DOI:10.1534/genetics.110.118695, FlyBase Page
26	m[1]	spontaneous	strong reduction of wing size	DOI:10.7717/peerj.12175, DOI:10.1016/j.devcel.2009.11.009
27	m[74f]	ethyl methanesulfonate	abnormally long wings	FlyBase page, Craymer, L. (1980). [New mutants report.] D. I. S. 55(): 197--200.
28	oc[1]	X ray	lack ocelli and associated bristles, underdeveloped brain, female sterile when homozygous	DOI:10.1093/genetics/139.4.1623, DOI:10.1007/s004270100149, DOI:10.1007/s003590050387
29	p[p]	spontaneous	no known physiological effects other than eye color	
30	pn[1]	spontaneous	defects in nervous system development, increased mortality	DOI:10.3389/fncel.2019.00076,
31	pr[1]	spontaneous	no known physiological effects other than eye color	
32	px[1]	spontaneous	extra wing veins, structural defects in wing veins	DOI: 10.1242/dev.126.23.5207
33	ras[2]	spontaneous	dark ruby eyes	DOI: 10.1007/BF00285747
34	ras[4]	spontaneous	dark ruby eyes, female sterility	DOI:10.1007/BF02191716
35	rb[1]	spontaneous	shortened male lifespan, defective eye pigment granules, reduced vision, behavior abnormalities	DOI: 10.1534/genetics.106.065011, DOI: 10.1007/pl00008688

36	ru[1]	spontaneous	heart abnormalities, misrotation of ommatidia, rough eyes	DOI:10.1371/journal.pgen.1000969, DOI:10.1016/j.devcel.2004.09.001, PMID: 10887159
37	sc[1]	spontaneous	loss of bristles on multiple body parts, loss of chemoreceptors on wing margins	DOI: 10.1007/BF00260865, DOI:10.1093/genetics/131.2.353
38	sd[1]	X ray	jagged wings, defects in bristles and halteres	DOI:10.1007/s004270050201, DOI:10.7554/eLife.00999, FlyBase page
39	sn[3]	spontaneous	deformed sensory bristles, abnormal axon morphology (curled)	DOI10.1242/dev.036517, DOI:10.1523/JNEUROSCI.2106-06.2006
40	speck[1]	spontaneous	no known defects besides darkening of wing hinge	
41	sr[1]	spontaneous	flightless, muscular and cuticular defects	DOI:10.1093/genetics/119.1.105, DOI: 10.1073/pnas.92.22.10344
42	st[1]	spontaneous	reduced lifespan and reduced locomotor ability	DOI:10.1242/jcs.216697
43	sv[de]	spontaneous	sterile, sensory defects, uncoordinated	ISBN:0070-7333, DOI:10.1242/dev.126.10.2261
44	Ubx[bx-1]	spontaneous	Halteres are transformed into wings as well as several other limb defects	DOI: 10.1002/j.1460-2075.1986.tb04497.x
45	Df(1)os-o(upd1[os-o],upd3[os-o])	X ray	reduced eye size, abnormal haltere position	DOI:10.1016/j.ydbio.2014.09.015
46	Ab(1)os-s(upd1[os-s],upd3[os-s])	spontaneous	no known deleterious effects, improved immune response to bacteria	DOI: 10.1038/ncomms14642
47	v[1]	spontaneous	abnormal, slow heart beat	DOI:10.1002/jez.2057
48	w[1]	spontaneous	decreased copulation rate	DOI:10.1371/journal.pone.0001391, DOI:10.1038/s41598-017-08155-y
49	w[1118]	spontaneous	deterioration of climbing ability, reduced courtship, retinal degradation under certain light conditions	DOI:10.3390/ijms222312967, DOI:10.1371/journal.pone.0077904, DOI:10.1091/mbc.E09-10-0917,DOI:10.3390/ijms222312967
50	y[1]	spontaneous	reduced mating success in males	DOI:10.7554/eLife.49388, DOI:10.1534/genetics.105.045666
